# Transcription termination and antitermination are critical for the fitness and function of the integrative and conjugative element Tn*916*

**DOI:** 10.1371/journal.pgen.1011417

**Published:** 2024-12-09

**Authors:** Erika S. Wirachman, Alan D. Grossman

**Affiliations:** Department of Biology, Massachusetts Institute of Technology, Cambridge, Massachusetts, United States of America; Norwegian University of Life Sciences: Norges miljo- og biovitenskapelige universitet, NORWAY

## Abstract

Premature expression of genes in mobile genetic elements can be detrimental to their bacterial hosts. Tn*916*, the founding member of a large family of integrative and conjugative elements (ICEs; aka conjugative transposons), confers tetracycline-resistance and is found in several Gram-positive bacterial species. We identified a transcription terminator near one end of Tn*916* that functions as an insulator that prevents expression of element genes when Tn*916* is integrated downstream from an active host promoter. The terminator blocked expression of Tn*916* genes needed for unwinding and rolling circle replication of the element DNA, and loss of the terminator caused a fitness defect for the host cells. Further, we identified an element-encoded antiterminator (named *canT* for conjugation-associated antitermination) that is essential for transcription of Tn*916* genes after excision of the element from the host chromosome. We found that the antiterminator is orientation-specific, functions with heterologous promoters and terminators, is processive and is most likely a *cis*-acting RNA. Insulating gene expression in conjugative elements that are integrated in the chromosome is likely a key feature of the interplay between mobile genetic elements and their hosts and appears to be critical for the function and evolution of the large family of Tn*916*-like elements.

## Author summary

Horizontal gene transfer allows bacteria to rapidly acquire new traits that can enhance their adaptability to different conditions. Integrative and conjugative elements (ICEs) are mobile genetic elements that reside integrated in a bacterial chromosome and can transfer to another cell via cell-to-cell contact through the element-encoded secretion system. ICEs often confer beneficial traits to their hosts, including antibiotic resistances, symbiotic/pathogenic determinants, metabolic capabilities, and anti-phage defense systems. Tn*916*, the first-described ICE, was identified based on its ability to transfer tetracycline resistance in the pathogen *Enterococcus faecalis*, and is found in several Gram-positive species. Once transferred into a new cell, Tn*916* integrates into AT-rich sequences, sometimes downstream from a host promoter. We found that Tn*916* has a transcription terminator near one end of the element that blocks transcription from an upstream host promoter, thereby protecting cells from detrimental effects of premature expression of element genes. Further, we found that Tn*916* has a transcription antitermination system that is essential for expression of element genes after excision from the host chromosome. Our findings highlight the complex layers of transcriptional regulation that have evolved in ICEs, impacting host cell viability and the spread of the element.

## Introduction

**Fig 1:**
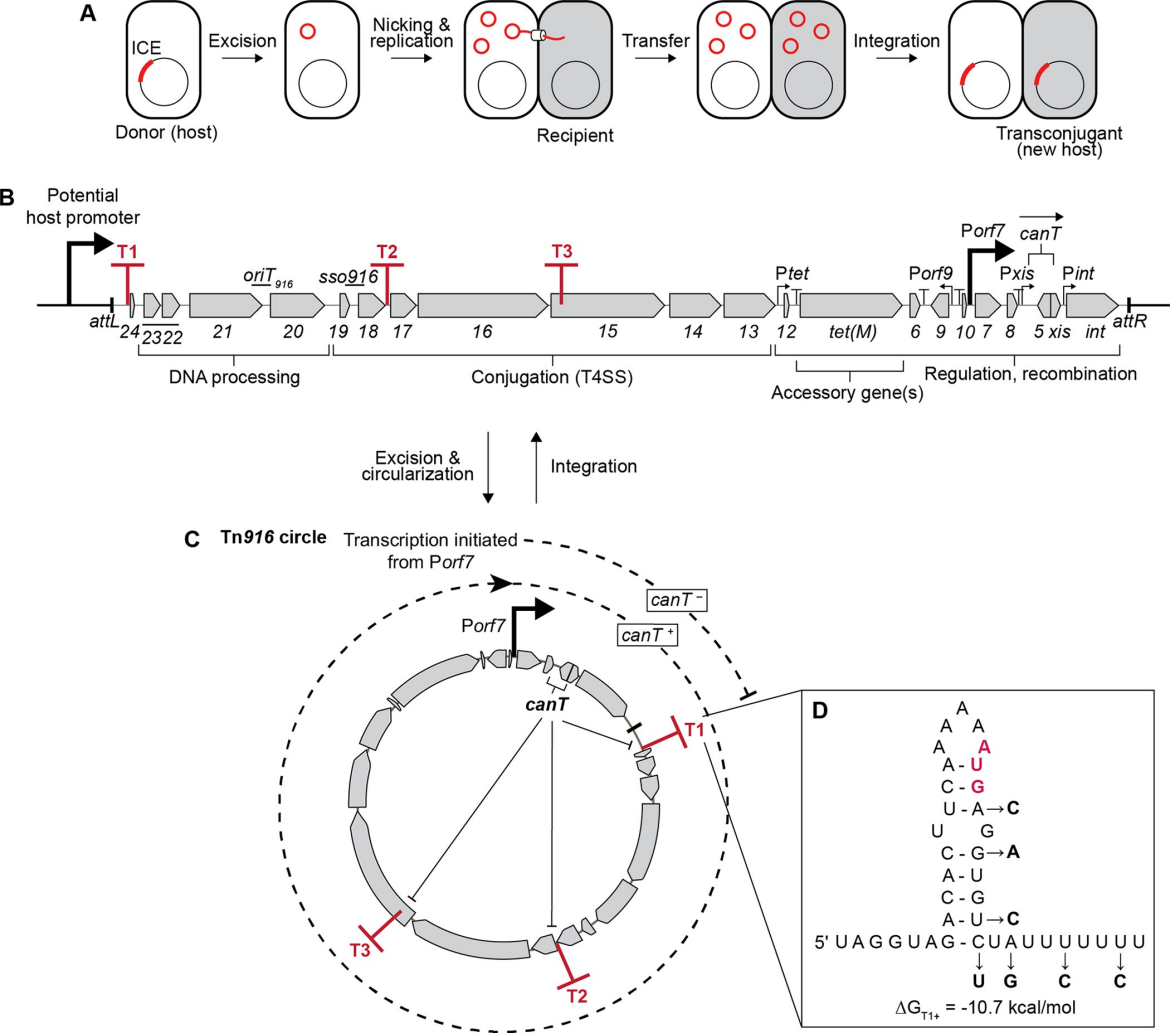
Genetic map of Tn*916* and its regulation. **A)** ICE life cycle. The chromosome is depicted as a circle with an ICE indicated in red. After excision, the circular double stranded ICE DNA (red) undergoes rolling circle replication. Transfer of linear ssDNA occurs to a recipient cell (shaded). Once transferred, the linear ssDNA is circularized and replicated to become dsDNA and then undergoes rolling circle replication and can be integrated to generate a stable transconjugant. **B)** Genetic map of Tn*916*. Gene names (numbers) are indicated below the corresponding genes. The ends of Tn*916* are indicated by black lines. Promoters are indicated by bent arrows. P*xis* is a weak promoter with poorly conserved -35 and -10 regions [19]. The origin of transfer (*oriT*_*916*_) [75], single strand origin of replication (*sso916*) [74], and the antiterminator *canT* are indicated above the map. The orientation of *canT* is indicated by the arrow above it. Three regions containing transcription terminators (T1, T2 consisting of T2a and T2b, T3) are indicated as red “T’s”. Functional modules of genes are indicated by brackets below the map. **C)** The excised circular form of Tn*916*. Following excision, *canT* is now upstream from the element genes needed for DNA processing and conjugation. P*orf7*, the main Tn*916* promoter that drives expression of the element genes [19] also drives production of *canT* RNA and the *canT* RNA inhibits termination at T1, T2 and T3, thereby allowing transcription of the DNA processing and conjugation genes (*orf24*-*orf13*). **D)** RNA sequence of terminator T1. The nucleotide positions of the base of the terminator stem were determined by the ARNold web server [28,29]. The minimum free energy of folding ΔG (of the stem-loop) was calculated using the RNAfold web server [31]. The bolded red AUG at the 3’ end of the loop indicates the start codon of *orf24*. The changes in the T1 mutant are indicated and were made to preserve the potential ribosome binding site and amino acid sequence of *orf24*.

Horizontal gene transfer helps drive microbial evolution, allowing bacteria to rapidly acquire new genes that can enhance their ability to thrive in different conditions. Integrative and conjugative elements (ICEs) are mobile genetic elements that normally reside integrated in a bacterial chromosome and can transfer to another cell via conjugation (Fig 1A). Once transferred, an ICE can integrate into the chromosome of the new host generating a transconjugant. ICEs often carry cargo genes that confer beneficial traits to their hosts, including antibiotic resistances, symbiotic/pathogenic determinants, metabolic capabilities, anti-phage defense systems, and many others [1–3]. While integrated in the host chromosome, cargo genes are often expressed, but most of the genes needed for the ICE lifecycle are not.

Tn*916* (~18 kb) (Fig 1B), the first-described ICE, was identified based on its ability to transfer tetracycline resistance in the pathogen *Enterococcus faecalis* [4,5]. Tn*916* and its relatives are found in many Gram-positive species, including *Enterococcus*, *Streptococcus*, *Staphylococcus*, and *Clostridium* [4–12], and function quite well in *Bacillus subtilis* [13–18]. As with other ICEs, Tn*916* contains genes needed for its lifecycle: recombination (integration, excision); DNA processing (nicking, unwinding, and rolling circle replication); conjugation (a type IV secretion system); and regulation. When Tn*916* is integrated in the chromosome, its DNA processing and conjugation genes are not expressed, largely due to the absence of a promoter within the integrated element (Fig 1B). After excision (circularization) of the element, the DNA processing and conjugation genes are expressed from the promoter for *orf7* (P*orf7*) (Fig 1C) [19].

Tn*916* integrates into AT-rich genomic regions [20–23] and these can be downstream from a host promoter. Integration in these regions is not random and when analyzed, there are hotspots for integration, and in some cases (e.g., *Clostridium difficile* CD37) there might be a unique integration site [24,25]. If Tn*916* genes are co-directional with a host promoter, then this promoter might drive expression of Tn*916* replication and conjugation genes (Fig 1B). Based on analogy to ICE*Bs1* from *B*. *subtilis* [26,27], we postulated that expression of these genes when the element is integrated would be detrimental to host cells, and that Tn*916* might have a mechanism to prevent this.

Here, we describe a transcription terminator (T1) near the left end of Tn*916* (Fig 1B) that is important for the fitness of host cells by functioning as an insulator to prevent transcription of element genes when Tn*916* is integrated downstream from a host promoter. We also discovered a region in Tn*916*, *canT* (conjugation-associated antiterminator) that is downstream from P*orf7* (Fig 1B and 1C), the promoter required for expression of the genes needed for conjugation [19]. *canT* prevents termination at element terminators and allows transcription of the DNA processing and conjugation genes (*orfs23-13*), but only after excision of Tn*916* from the host chromosome. *canT* is essential for horizontal transfer of Tn*916*.

## Results

### Identification of functional transcription terminators in Tn*916*

We identified four predicted intrinsic transcription terminators (T1, T2a, T2b, T3) between the left end through the conjugation genes of Tn*916* (Fig 1B) using online tools that predict RNA secondary structure and possible intrinsic terminators [28–31]. Terminator T1 is upstream of *orf24*, near the left end of Tn*916* (Fig 1B and 1D). T2a and T2b (indicated as T2), are adjacent to each other and downstream of *orf18* (Figs 1B and S1A) and T3 is within *orf15* (Figs 1B and S1B). Because of its location near the left end of Tn*916*, we focused on T1 as a potential insulator of element gene expression when Tn*916* is downstream from a host promoter.

**Fig 2:**
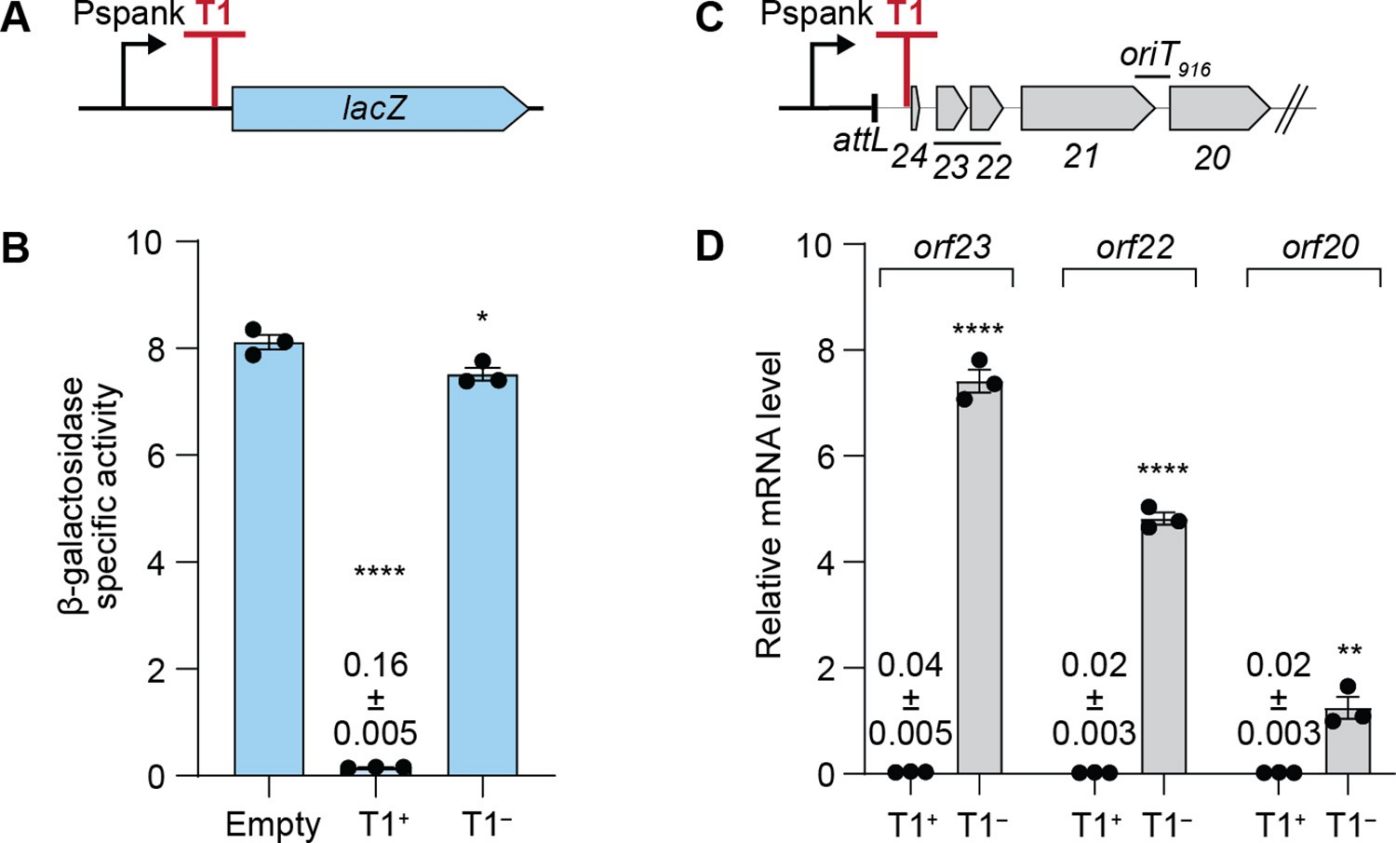
Terminator T1 of Tn*916* is functional within and outside of Tn*916*. **A)** Schematic of *lacZ* reporter construct with terminator T1 between Pspank and *lacZ*. **B)** β-galactosidase specific activities of the *lacZ* reporters with and without a functional terminator T1, either T1 was absent (Empty, ESW252), intact (T1^+^, ESW605), or mutated (T1^–^, ESW606), were measured two hours after induction of Pspank with IPTG. Data presented are averages from three independent experiments with error bars depicting standard error of the mean (mean ± SEM). P-values were calculated by an ordinary one-way ANOVA followed by Dunnett’s multiple comparisons test (* *P*<0.05, **** *P*<0.0001) using the GraphPad Prism version 10. Significance comparisons were made against the strain without T1 (Empty). **C)** Schematic of Pspank-Tn*916*. Full-length Tn*916* is present, but only *orf24* through *orf20* are indicated in the schematic. **D)** Relative mRNA levels of the indicated genes from Pspank-Tn*916* with an intact (T1+, ESW179) or mutant (T1–, ESW247) terminator T1 were measured one hour after induction of Pspank with IPTG. Data presented are from three independent experiments with error bars depicting standard error of the mean (mean ± SEM). P-values were calculated by two-tailed *t*-test (** *P*<0.01, **** *P*<0.0001) using the GraphPad Prism version 10. Significance comparisons were made for the amount of mRNA from each gene comparing the terminator mutant (T1^–^) to that from the strain with a functional terminator T1 (T1^+^).

To test its efficiency, we cloned T1 between the IPTG-inducible promoter Pspank and *lacZ* at an ectopic chromosomal locus, outside of Tn*916* (Fig 2A) in *Bacillus subtilis*. In the presence of T1, expression of *lacZ* was reduced to <5% of that in the absence of T1 (Fig 2B). Mutations in T1 (T1^–^) that disrupted the stem and the U-tract of the terminator (Fig 1D) restored expression of *lacZ* (Fig 2B), indicating that T1 is a functional terminator with an efficiency of >95%.

### T1 functions to insulate genes in Tn*916* from readthrough transcription from a promoter in the host chromosome

To test the ability of terminator T1 to insulate transcription of Tn*916* genes from an upstream promoter in the chromosome, we cloned Pspank upstream of Tn*916* with an intact or mutant T1 (Fig 2C) and measured the amount of mRNA of three Tn*916* genes (*orf23*, *orf22*, and *orf20*) located downstream of T1 by RT-qPCR. Expression of Tn*916* genes was coming solely from the integrated element because the Pspank insertion prevented excision (S2 Fig), likely by altering the region just upstream of the junction between the chromosome and Tn*916* that Int is known to bind [32]. After induction of Pspank (addition of IPTG), there were low levels of mRNA from the three genes in wild-type (T1^+^) Tn*916* (Fig 2D). In contrast, the T1 mutant (T1^–^) had elevated amounts of mRNA from each of the three genes (Fig 2D). Based on these results, we conclude that T1 is a functional terminator that greatly reduces transcription into Tn*916* from an upstream promoter in the chromosome.

### Predicting an antitermination mechanism in Tn*916*

Transcription of Tn*916* genes needed for conjugation is normally driven by P*orf7* after excision and circularization of the element from the chromosome [19] (Fig 1C). Because terminator T1 is between the promoter P*orf7* and conjugation genes after the element excises and is in the circular form, we postulated that there would be an antitermination mechanism to enable transcription of the Tn*916* genes essential for conjugative transfer. Previous work found that a transposon (Tn*5*) insertion in codon 41 (of 83 total codons) of the predicted gene *orf5* eliminated conjugation, leading to the inference that *orf5* might encode a protein essential for conjugation [33,34]. However, no transcripts from the putative *orf5* have been detected [19,35]. Experiments described below demonstrate that the putative *orf5* protein product is not required for expression of Tn*916* genes or for conjugation. Rather, there is an overlapping sequence now called *canT* (conjugation-associated antitermination) that functions in the opposite orientation from the putative *orf5* and is essential for transcription antitermination and conjugation following excision of the Tn*916* from the chromosome (Fig 1C).

### The putative gene *orf5* does not encode a protein product necessary for conjugation

**Fig 3:**
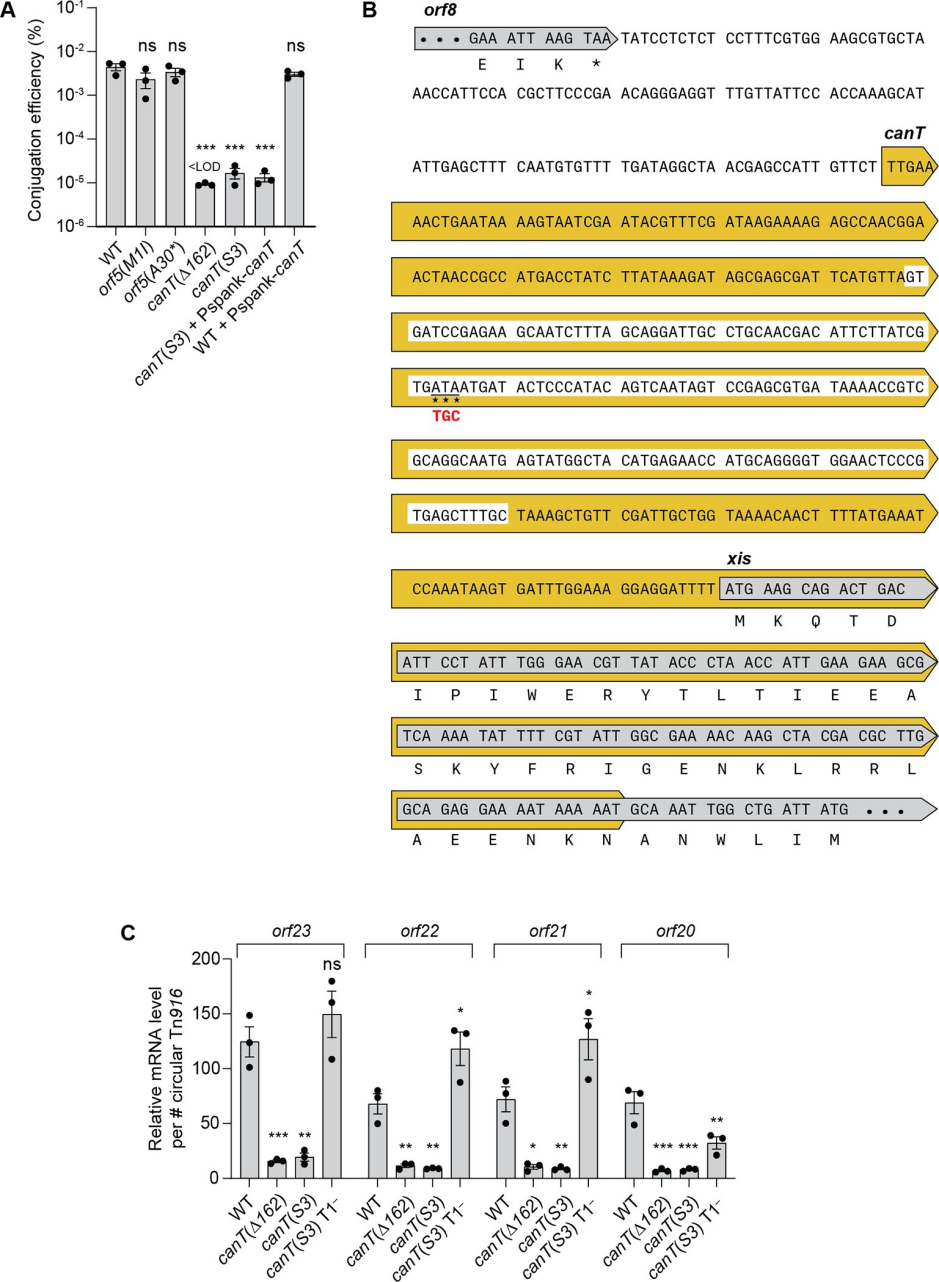
Phenotypes caused by mutations in *canT* and the putative *orf5*. **A)** Conjugation efficiencies. Donors contained wild-type Tn*916* (WT, CMJ253), Tn*916* with a mutation in the start codon of the putative *orf5* [*orf5*(*M1I*), ESW104], Tn*916* with a premature stop codon in the putative *orf5* [*orf5*(A30*), ESW420], Tn*916* with a deletion [*canT*(Δ*162*), ESW120] or 3 bp changes [*canT*(*S3*), ESW98] in *canT*, the Tn*916 canT*(*S3*) mutant with wild-type *canT* expressed in *trans* [*canT*(*S3*)+Pspank-*canT*, ESW771], and wild-type Tn*916* with wild-type *canT* expressed in *trans* [WT+Pspank-*canT*, ESW781]. Conjugation efficiencies were calculated as the number of transconjugants per input donor. Data presented are from three independent experiments with error bars depicting standard error of the mean (mean ± SEM). All three independent mating assays of *canT*(Δ*162*) resulted in conjugation efficiencies that are below the limit of detection. P-values were calculated by an ordinary one-way ANOVA followed by Dunnett’s multiple comparisons test (*** *P*<0.001) using the GraphPad Prism version 10. Significance comparisons were made against the WT strain. **B)** Schematic of *canT* alleles. Parts of the 3’ end of *orf8* and the 5’ end of *xis* are indicated inside of gray arrows. The 452-nucleotide *canT* region is shown inside of the yellow arrow. The region deleted (162 nucleotides) in *canT*(Δ*162*) is highlighted in white. The nucleotides changed in *canT*(*S3*) are underlined and indicated with stars, with the mutant sequence directly below the wild-type sequence. Prior to determining that the putative *orf5* is not relevant as an open reading frame, we made the *S3* mutation that would change the predicted tyrosine residue that was predicted computationally to be a potential site for phosphorylation. **C)** Relative mRNA levels normalized to the number of circular Tn*916* per cell. Tn*916* alleles include: wild-type (WT, CMJ253), *canT*(Δ*162*) (ESW120), *canT*(*S3*) (ESW98), *canT* and terminator T1 double mutant [*canT*(*S3*) T1^–^, ESW630]. Data presented are from three independent experiments with error bars depicting standard error of the mean (mean ± SEM). P-values were calculated by an ordinary one-way ANOVA followed by Dunnett’s multiple comparisons test (* *P*<0.5, ** *P*<0.01, *** *P*<0.001) using the GraphPad Prism version 10. Significance comparisons were made against the WT strain for each gene.

We made two different mutations that should prevent the production of an *orf5*-encoded protein. 1) We changed the predicted start codon from AUG to AUA [*orf5*(*M1I*)], which should prevent translation of *orf5* but preserve the amino acid sequence of the overlapping *xis*. 2) We changed codon 30 (of 83 total) to a stop codon [GCA to UAA, *orf5*(A30*)]. In both cases, the conjugation efficiency of Tn*916* was indistinguishable from that of an otherwise wild-type element (Fig 3A). These results indicate that the role of the putative *orf5* in conjugation is not as a protein-coding gene. More likely, the initial Tn*5* insertion in the putative *orf5* [33,34] disrupted a region that overlaps *orf5* and this region is required for conjugation and likely functions as RNA.

### Identification of a region in Tn*916* that is required for transcription of conjugation genes

We found that the region between the 3’ end of *orf8* and *xis* was required for conjugation and expression of several Tn*916* genes. A deletion that removed 162 bp [*canT*(*Δ162*)] (Fig 3B, white-highlighted) and a 3 bp substitution (ATA to TGC) [*canT*(*3S*)] (Fig 3B) both caused an approximately 100-fold drop in conjugation (Fig 3A). In contrast to the effect on conjugation, the excision frequency of the mutants was not decreased (WT: 1.68 ± 0.06%; *canT*(*Δ162*): 2.04 ± 0.05%; *canT*(*3S*): 1.82 ± 0.06%), indicating that the region altered in these mutants is normally required for conjugation but not excision, similar to the phenotypes described for the Tn*5* insertion in the putative *orf5* [33,34].

We also found that this region was required for expression of genes downstream from the terminator T1. We measured the amount of mRNA for *orf23*, *orf22*, *orf21*, and *orf20* per copy of circular Tn*916*, thereby normalizing for excision and copy number of the excised element. The amount of mRNA for these genes in the two different *canT* mutants was greatly reduced (Fig 3C), indicating that the normal function of *canT* is to enable expression of these genes. Together, these results show that *canT* affects expression of genes downstream from T1 in the Tn*916* circle, but is not required for excision.

If *canT* enabled expression of these genes by allowing transcription to read through T1 (as opposed, for example, to containing a strong promoter), then inactivation of T1 should restore gene expression in the absence of *canT*. Indeed, we found that loss of T1 restored expression of *orfs23*, *22*, *21*, and *20* in the *canT*(*3S*) mutant. Levels of mRNA from all four *orfs* were increased at least four-fold in the *canT*(*S3*) T1^–^ double mutant compared to the *canT*(*S3*) single mutant (Fig 3C). Based on these results, we conclude that *canT* functions as an antiterminator in Tn*916*, enabling transcription to read through terminator T1.

### *canT* is sufficient for antitermination and functions processively and in *cis*

#### *canT* functions as an antiterminator in the absence of other Tn*916* genes

**Fig 4:**
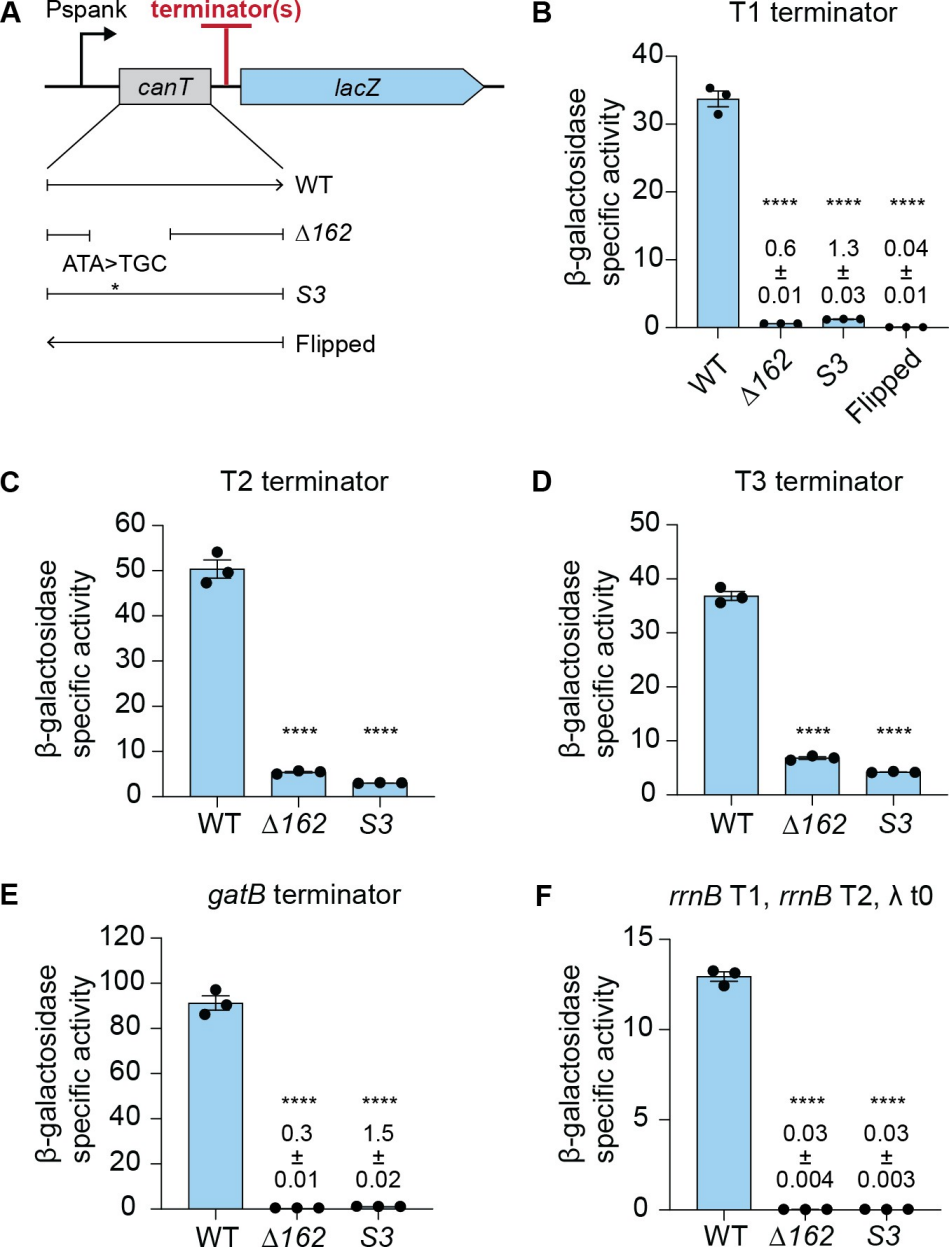
*canT* functions as an antiterminator without any other Tn*916* components. **A)** Schematic of *lacZ* reporter construct with *canT* alleles and terminator(s): T1 (**B**); T2 (**C**); T3 (**D**); *gatB* terminator (**E**); *rrnB* T1, *rrnB* T2, and λ t0 (**F**); between Pspank and *lacZ*. *canT* alleles included wild-type (WT), (Δ*162*), (*S3*), wild-type but in the opposite orientation (Flipped). **B-F)** β-galactosidase specific activities from the indicated strains following two hours induction of Pspank with IPTG. Data for each are from three independent experiments with error bars depicting standard error of the mean (mean ± SEM). P-values were calculated by an ordinary one-way ANOVA followed by Dunnett’s multiple comparisons test (**** *P*<0.0001) using the GraphPad Prism version 10. Significance comparisons were made against the WT strain. **B)** T1 from Tn*916* with *canT* alleles: WT (ESW398), Δ*162* (ESW408), *S3* (ESW407), Flipped (ESW440). **C)** T2 from Tn*916* with *canT* alleles: WT (ESW492), Δ*162* (ESW481), *S3* (ESW623). **D)** T3 from Tn*916* with *canT* alleles: WT (ESW493), Δ*162* (ESW482), *S3* (ESW626). **E)** Terminator from *gatB* with *canT* alleles WT (ESW421), Δ*162* (ESW473), *S3* (ESW449). **F)** Three terminators, T1 and T2 from *E*. *coli rrnB*, and t0 from phage lambda with *canT* alleles WT (ESW783), Δ*162* (ESW785), *S3* (ESW784).

We cloned a 452 bp fragment (Fig 3B, yellow arrow) that contains wild-type or mutant *canT* between Pspank and T1 in the *lacZ* reporter described above (Figs 2A and 4A). Expression of *lacZ* with the *canT*(Δ*162*) and *canT*(*S3*) mutants was <5% of that with wild-type *canT* (Fig 4B). These results indicate that *canT* functions to allow transcription to read through T1, consistent with the results with an intact Tn*916*.

#### *canT* functions in *cis*

We wished to determine if there was a *trans*-acting factor that was disrupted in the *canT* mutant, or if *canT* functioned in *cis*. We found that a functional *canT* in the context of the reporter described above was unable to complement a Tn*916 canT* mutant. That is, there was no detectable conjugation of the Tn*916 canT* mutant in strains with a functional *canT* located elsewhere in the chromosome (Fig 3A, *canT*(*S3*) + Pspank-*canT*). Additionally, conjugation of wild type Tn*916* (*canT*+) was normal in strains with the functional *canT* located elsewhere, indicating that expression of *canT* in *trans* did not inhibit its activity in the native context (Fig 3A, WT + Pspank-*canT*). Based on the results above, we conclude that *canT* acts in *cis* to cause transcription antitermination.

We also found that *canT* function is orientation-specific, consistent with an antiterminator that functions as RNA. We cloned the 452 bp fragment that contains wild-type *canT* in the opposite (flipped) orientation from what is normally found in Tn*916* (Fig 4A). There was little to no expression of *lacZ* from the reporter (Fig 4B), indicating that *canT* function is orientation-specific. We note that clones with wild-type *canT* in each orientation contain the putative *orf5*, which overlaps *canT*. In the construct with the opposite orientation, the putative *orf5* is oriented in the sense direction with Pspank and hence *orf5* should be transcribed. These findings reinforce the conclusions above that if *orf5* encodes a protein product, it is not involved in antitermination. Based on these results, we conclude that *canT* functions as an antiterminator, is orientation-specific, no other Tn*916* genes are needed for its function, and that it is most likely active as RNA.

In attempts to identify smaller fragments that retained *canT* function, we cloned fragments of varying lengths into the reporter (S3 Fig), as described for the 452 bp fragment. We found that a 400 bp fragment had antitermination activity (S3C Fig**)**. However, the *canT*(*S3*) mutation in this fragment had little or no effect on antitermination activity (S3C Fig**)**. In other words, the properties of the 400 bp fragment did not mimic those of the intact Tn*916* (Fig 3C) or the 452 bp fragment in the reporter (Figs 4 and S3B). Some shorter DNA fragments also had antiterminator activity (e.g., S3D, S3E and S3G Fig), although we did not test the effects of the *canT*(*S3*) mutation on these fragments. Based on these results, we decided to use the 452 bp fragment in further experiments as it provided high antitermination activity and accurately reflected the effects of the *canT*(*S3*) mutation observed in the intact Tn*916*. Below, we also describe the predicted secondary structures of some of the fragments with antitermination activity.

#### *canT* functions as an antiterminator for T2 and T3 of Tn*916*

T2 and T3 were cloned individually into the *canT*-*lacZ* reporters in place of T1 (Fig 4A). Similar to results with T1, expression of *lacZ* was high in the presence of wild-type *canT* and much lower with the mutant *canT* (Fig 4C and 4D).

#### *canT* functions as an antiterminator for heterologous terminators and is processive

We cloned the terminator located downstream of *B*. *subtilis gatB* into the *canT*-*lacZ* reporters (Fig 4A). Expression of *lacZ* was high with wild-type *canT* and quite low with the *canT* mutants (Fig 4E). We also cloned three terminators, the two from *E*. *coli rrnB* (*rrnB* T1, *rrnB* T2) and the phage lambda terminator t0, between *canT* and *lacZ* (Fig 4A). There was expression of *lacZ* with wild-type *canT*, but no detectable expression with the *canT* mutants (Fig 4F). Together, our results indicate that *canT* functions on T1, T2, and T3 of Tn*916*, acts on heterologous terminators, functions in *cis*, and acts processively.

#### Predicted secondary structures of *canT* RNA

Based on the results above, it is most likely that *canT* RNA functions an antiterminator, analogous to other RNA antiterminators [36–41]. The known RNA antiterminators all contain predicted stem-loop structures and are sometimes complicated [36–41]. We used the ViennaRNA package [30] to predict *canT* RNA secondary structures for the wild-type 452-nucleotide sequence (S4A Fig) and three different variants that are inactive (S4B–S4D Fig) and one variant that is active (S4E Fig).

The 452-nucleotide sequence that has *canT* antitermination activity is predicted to fold into a complex structure with multiple regions of base pairing and stem-loops (S4A Fig). The left and right parts (as drawn in S4A Fig, green and blue boxes, respectively) are each predicted to have at least three complex stem-loops. Both the *canT*(Δ*162*) (S4B Fig) and *canT*(*S3*) (S4C Fig) mutants of the 452-nucleotide *canT* were defective in antitermination and were missing structures on the left side of the wild-type *canT* (S4A Fig, green box), perhaps indicating that these structures play a role in antitermination. However, the predicted secondary structure of the antitermination-defective 291-nucleotide sequence (S3F and S4D Figs) contains the sequences that are missing or altered in the *canT*(*Δ162*) and *canT*(*S3*) mutants. This indicates that this predicted structure (S4D Fig, green box) is not sufficient to cause antitermination. Additionally, a 341-nucleotide sequence that has antitermination activity (S3G Fig) is predicted to have parts of the predicted secondary structures (S4E Fig) that are also predicted to be present in sequences that are not active (S4B–S4D Fig). Based on these observations, we conclude that interpreting antitermination activity from secondary structure predictions of *canT* RNA is complicated. We suspect that whether or not the secondary structure predications are accurate, there is an RNA tertiary structure that is essential for antitermination activity. We have not further pursued sequence or structural analyses of this region.

### Terminator T1 confers a fitness benefit to cells with Tn*916* integrated downstream from a strong promoter

**Fig 5:**
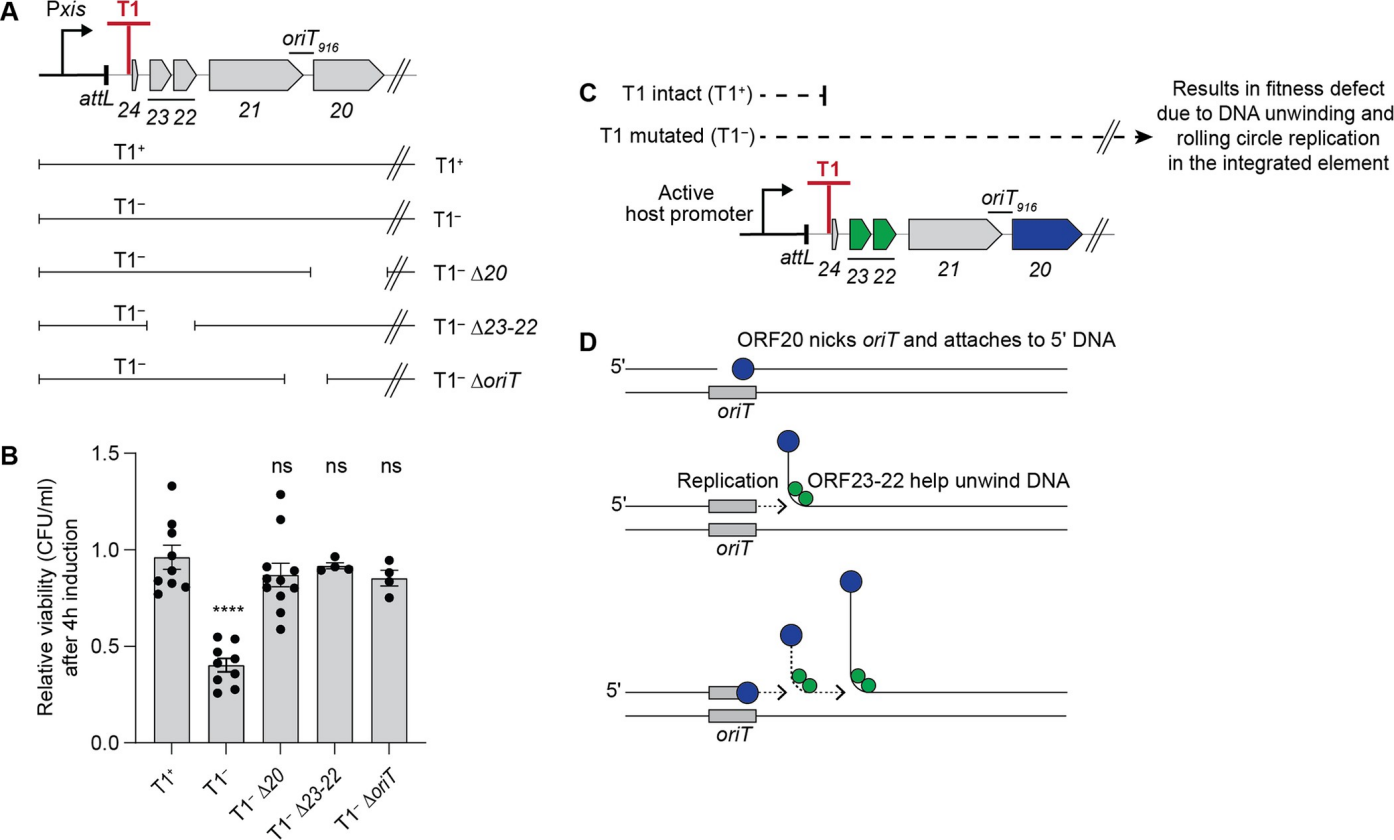
Rolling circle replication causes a fitness defect in Tn*916* insertions downstream of an active host promoter. **A)** Schematic of P*xis*-Tn*916* either with intact (T1^+^, ESW270) or mutant (T1^–^, ESW271) terminator T1, or with a mutant terminator combined with loss-of-function mutations in *orf20* (T1^–^ Δ*20*, ESW316), *orf23-22* (T1^–^ Δ*23–22*, ESW363), *oriT* (T1^–^ Δ*oriT*, ESW414). Full-length Tn*916* is present, but only *orf24* until *orf20* are indicated in the schematic. P*xis* used here is the strong promoter from ICE*Bs1* that is repressed by the ICE*Bs1* repressor ImmR. ImmR is inactivated by the metalloprotease ImmA following expression of RapI [42–44]. *rapI* was expressed from a xylose-inducible promoter (Pxyl-*rapI*) following addition of xylose to cells. **B)** Relative viability of P*xis*-Tn*916* strains shown in (**A**) was measured four hours after induction of P*xis* and calculated as the number of CFUs following induction, divided by that in the uninduced culture grown in parallel (a value of “1” indicates there is no change in CFUs with induction). Data presented are from at least four independent experiments with error bars depicting standard error of the mean (mean ± SEM). P-values were calculated by an ordinary one-way ANOVA followed by Dunnett’s multiple comparisons test (**** *P*<0.0001) using the GraphPad Prism version 10. Significance comparisons were made against the T1^*+*^ strain. **C)** Schematic of Tn*916* gene transcription when the element is inserted downstream of a host promoter. Genes necessary for DNA replication, including *orf23-22* (green) and *orf20* (blue), are not transcribed when terminator T1 is intact (T1^+^), but are transcribed when T1 is mutated (T1^–^). Expression of these genes from integrated Tn*916* leads to DNA unwinding and rolling circle replication occurring in the integrated element, which causes a fitness defect to the host cells. **D)** Cartoon of repeated rolling circle replication from the Tn*916 oriT* that is integrated in the chromosome (modified from [26]). The relaxase ORF20 (blue circles) nicks the origin of transfer, *oriT* (gray bar), that also functions as an origin of replication [18] and is covalently attached to the 5’ end of the DNA. Replication extends (dotted line with arrow) from the free 3’-OH and regenerates a functional *oriT* that is a substrate for nicking by another molecule of the relaxase. ORF23-22 (green circles) act as helicase processivity factors, helping the host-encoded helicase, PcrA (not drawn), unwind the DNA [18,45]. The rest of the replication machinery (not drawn) is composed of host-encoded proteins.

We found that T1 of Tn*916* conferred a fitness benefit to the host when Tn*916* was downstream from and co-directional with a strong promoter in the host chromosome. Initially, we inserted the strong inducible promoter P*xis* from ICE*Bs1* upstream from Tn*916* (Fig 5A). P*xis* is repressed by ImmR and is derepressed following cleavage of ImmR by the protease ImmA which is activated by RapI [42–44]. We induced transcription from P*xis* by expressing *rapI* from a xylose-inducible promoter (Pxyl-*rapI*) (Methods). Transcription from P*xis* had little or no effect on the cell viability when Tn*916* contained a functional T1. That is, the number of viable cells four hours after derepression of P*xis* (the addition of xylose to induce Pxyl-*rapI*) was virtually the same as in cells grown similarly but without xylose (Fig 5B). In contrast, in cells with a mutant T1 (T1^-^), there was a two-fold reduction in CFUs after four hours of expression from P*xis* (+xylose) compared to no expression (no xylose) (Fig 5B). Based on these results, we conclude that terminator T1 is important for the fitness of host cells that contain Tn*916* downstream from a strong promoter.

By analogy to findings with ICE*Bs1* [26,27], we suspected that the decrease in CFUs of cells with the Tn*916* T1 mutant was due to autonomous rolling circle replication of Tn*916* that remained integrated in the chromosome. Tn*916* has three genes (*orf23*, *22*, *20*) and a site (*oriT*) that are required for DNA unwinding, autonomous rolling circle replication [18], and conjugation. *orf20* encodes the relaxase that nicks the element at the origin of transfer, *oriT* (that also functions as an origin of replication), to initiate DNA unwinding and rolling circle replication. *orf22* and *orf23* both encode helicase processivity factors that help the host-encoded helicase PcrA unwind the element DNA after nicking [18,45]. Deletions of *orf20*, *orf23-22*, or *oriT* all alleviated the fitness defect caused by expression from P*xis* into Tn*916* in the absence of a functional T1 (Fig 5A and 5B). Based on these results, we conclude that the terminator T1 in Tn*916* is important for preventing expression of the DNA unwinding and replication genes in Tn*916* when the element is downstream from a strong promoter and that loss of this terminator results in a fitness defect that is due to DNA unwinding and-or autonomous rolling circle replication of Tn*916* while it is in the host chromosome (Fig 5C and 5D).

### Isolating cells with Tn*916* integrated downstream of a host promoter

**Fig 6:**
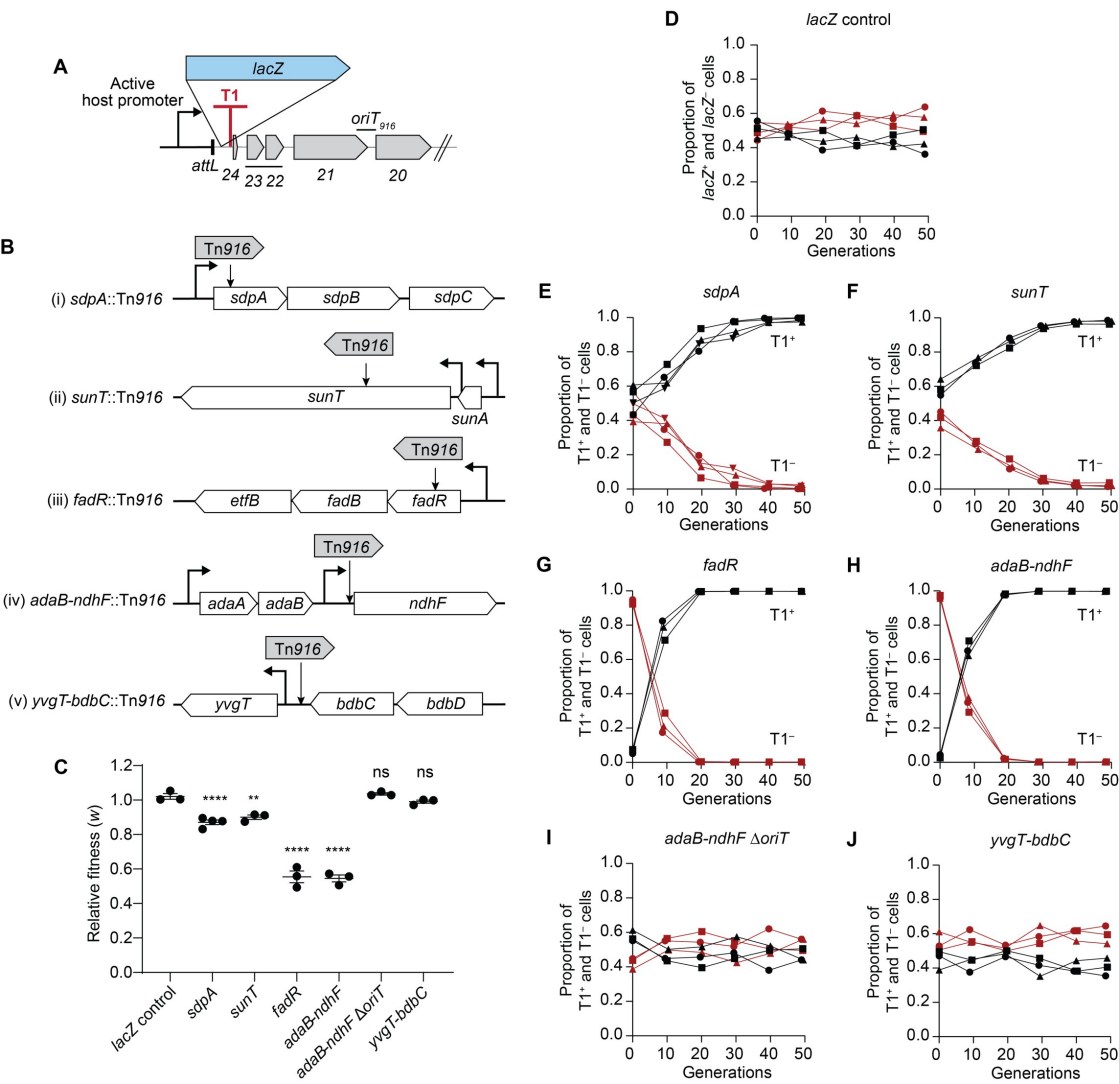
Terminator T1 confers a fitness benefit in Tn*916* insertions downstream of an active host promoter. **A)** Schematic of Tn*916*-*lacZ* integrated downstream from an active host promoter. *lacZ* was cloned within Tn*916*, upstream of terminator T1 such that *lacZ* is expressed when Tn*916* integrates downstream of an active host promoter. Tn*916-lacZ* (not downstream from an active promoter, so phenotypically LacZ–) in strain (ESW261) was used as a donor used to isolate Tn*916* insertions in transconjugants that are downstream of an active host promoter. **B)** Schematic and maps of the Tn*916* insertions used for pairwise competition assays. Tn*916* integrated at *sdpA* (i), *sunT* (ii), *fadR* (iii), and *adaB*-*ndhF* (iv) are located downstream of active promoters. Tn*916* at *yvgT-bdbC* (v) is oriented opposite the direction of transcription of the host genes. **C)** Fitness of T1^–^ cells relative to T1^+^ cells calculated as Malthusian parameters ratio of T1^–^ to T1^+^ (Methods). Data presented from at least three independent experiments with error bars depicting standard error of the mean (mean ± SEM). P-values were calculated by an ordinary one-way ANOVA followed by Dunnett’s multiple comparisons test (**P<0.01, ****P<0.0001) using the GraphPad Prism version 10. Significance comparisons were made against the *lacZ* control. **D-J)** Population dynamics over the course of the pairwise competition assays. The initial ratio of *lacZ*^+^ and *lacZ*^−^cells was ~1:1 for (**D**). The initial ratio of T1^+^ to T1^–^ cells was ~1:1 for (**E**,**F**,**I**,**J**) and ~1:10 for (**G**,**H**). At least three independent experiments were done for each competition and individual data points are shown. The x-axis (generations) represents the number of population doublings. Circles, replicate 1; squares, replicate 2; triangles, replicate 3; inverted triangles, replicate 4. Black, T1^+^; red, T1^–^. Competition assay results of *lacZ*^+^ and *lacZ*^−^cells: (**D**) (ESW617 vs ESW616). Competition assay results of T1^+^ vs T1^–^ cells: (**E**) *sdpA* (ESW675 vs ESW693), (**F**) *sunT* (ESW677 vs ESW695), (**G**) *fadR* (ESW724 vs ESW725), (**H**) *adaB-ndhF* (ESW722 vs ESW723), and (**J**) *yvgT-bdbC* (ESW713 vs ESW714). (**I**) T1^+^ vs T1^–^ Δ*oriT* cells of Tn*916* integrated at *adaB-ndhF* (ESW722 vs ESW728).

We wished to determine if T1 also contributed to host fitness when Tn*916* was integrated downstream from endogenous host promoters. To isolate Tn*916* insertions downstream from active host promoters, we cloned *lacZ* near the left end of Tn*916*, upstream of T1 (Tn*916-lacZ*) (Fig 6A) and used a strain with this element as a donor for conjugation. Transconjugants were selected on plates with tetracycline and X-gal and blue colonies were picked and verified for the presence of Tn*916* and expression of *lacZ*. Several strains were chosen for further analyses and the sites of integration were determined by arbitrary PCR and sequencing (Methods). Strains with an insertion in *sdpA*, *sunT*, *fadR*, and *adaB*-*ndhF* (Fig 6B) were chosen for further analyses. We removed *lacZ* from each insertion and then introduced mutations that inactivate terminator T1. As a control, we used an insertion in *yvgT-bdbC* in which the Tn*916* replication and conjugation genes were not co-directional with a host promoter (Fig 6B). We also used the initial Tn*916-lacZ* alleles (*lacZ*^+^ T1^+^) to monitor transcription from the host promoters.

### Fitness benefits of terminator T1 in Tn*916* insertions that are downstream from host promoters

We found that T1 contributed to host fitness when Tn*916* was integrated downstream from endogenous host promoters. To measure the fitness benefit conferred by T1, we did pairwise competition assays between strains with and without a functional T1 (T1^+^ versus T1^–^). The two strains were mixed in an appropriate ratio (~1:1 or ~1:10), spotted on LB agar for 24 hours, harvested, and then a dilution of the mix was spotted again on fresh LB agar and repeated for up to five days. The total cell number increased approximately 300- to 1,000-fold each 24-hour cycle, the equivalent of 8–10 cell doublings, and totaling approximately 50 cell doublings over five days. The proportion of each strain in the mixed population was determined every 24 hours, and the relative fitness (*w*) of T1^–^ to T1^+^ cells was calculated. Because *lacZ* had been removed from the Tn*916* insertions, we used *lacZ* located elsewhere in the chromosome as a fitness-neutral marker in T1^+^ cells (Fig 6C and 6D) to distinguish them from the T1^–^ cells.

We found that the loss of a functional T1 caused a fitness defect in the four different Tn*916* insertions that were downstream from a host promoter. The proportion of T1^–^ cells in the population dropped from ~50% to ~10% in ~30 doublings for both *sdpA*::Tn*916* and *sunT*::Tn*916* (Fig 6E and 6F), with mean relative fitness of 0.87 and 0.90, respectively (Fig 6C). The proportion of T1^–^ cells in the population dropped from ~90% to ~1% in ~20 doublings for both *fadR*::Tn*916* and *adaB-ndhF*::Tn*916* (Fig 6G and 6H), with mean relative fitness of 0.55 for both (Fig 6C). These results show that T1 can confer a fitness benefit to cells that contain Tn*916* downstream from and codirectional with a host promoter.

The fitness defect caused by the loss of T1 in the insertions was due to DNA unwinding and-or autonomous rolling circle replication. We deleted *oriT* from Tn*916* inserted in *adaB-ndhF*, thereby preventing both DNA unwinding and autonomous replication of Tn*916* and found that the proportion of T1^+^ and T1^–^ Δ*oriT* cells did not significantly change over the entire competition experiment (Fig 6I), indicating that the fitness defect caused by the loss of T1 was completely suppressed. The mean relative fitness was 1.04 (Fig 6C), in marked contrast to that of the element with a functional *oriT* (mean relative fitness of 0.55). These results are consistent with those described above for P*xis*-Tn*916* T1^–^ (Fig 5B).

The fitness effects caused by T1 were dependent on the presence of an upstream host promoter that was co-directional with Tn*916* genes. We used a Tn*916* insertion that is between *yvgT*-*bdbC* and oriented opposite the direction of transcription of the host genes (Fig 6B). There was no significant change in the proportion of cells with Tn*916* with T1^+^ versus T1^–^ during the entire competition (Fig 6J). The mean relative fitness was 0.99 (Fig 6C). Based on these results, we conclude that the terminator T1 of Tn*916* is important for the fitness of host cells when the element is downstream from and co-directional with an active host promoter, and that the primary function of T1 is to prevent autonomous rolling circle replication of Tn*916* that is in the host chromosome.

We tested whether the fitness effects might be correlated with apparent promoter strengths. We determined promoter activities by measuring β-galactosidase activity during growth in defined minimal liquid medium and after eight hours of growth as a spot on an LB agar plate using the initial Tn*916-lacZ* insertions described above (Fig 6A and 6B).

During exponential growth in defined minimal medium, *fadR* had the highest rate of transcription as measured by β-galactosidase synthesis, followed by *adaB-ndhF*, *sunT*, and *sdpA* (S5A Fig). This appears to roughly correlate with the relative effects on fitness in which Tn*916* expression from *fadR* and *adaB-ndhF* had larger defects than that from *sunT* and *sdpA* (Fig 6C). However, after eight hours of growth as spots on an LB agar plate (conditions that are closer to those used in the competition experiments to measure fitness), *sunT* appeared to have the highest expression, followed by *sdpA*, *fadR*, and *adaB-ndhF* (S5B Fig). The fitness effects observed during the competition experiments (Fig 6C) did not correspond to these levels of β-galactosidase synthesis. Additionally, we compared published transcriptomics data [46] for each of the four loci and the effects on fitness. The published data [46] indicate that during exponential growth, transition to stationary phase, and stationary phase in LB medium, *sunT* had the highest mRNA levels, followed by *fadR*, *sdpA*, and *adaB-ndhF*, again, not correlating or corresponding to the observed effects on fitness in our competition experiments (Fig 6C).

Based on the published information about transcription from the relevant promoters and our measurements, we believe that quantitatively different fitness effects caused by expression of Tn*916* genes from different host promoters are due to a combination of promoter strength, stability of the hybrid mRNAs (host gene and Tn*916* genes co-transcribed), and the ability of the hybrid mRNAs to be translated, all during complex and continually changing growth conditions in the mixed communities on a solid surface.

## Discussion

We found that Tn*916* has a transcription termination-antitermination system that is crucial for element function and host cell fitness. This system includes terminators T1, T2 (T2a + T2b), and T3 and the antiterminator *canT*. When Tn*916* integrates downstream from an active host promoter, T1 insulates expression of genes needed for unwinding and rolling circle replication of the element DNA and preserves host cell fitness. We suspect that the function of terminators T2 and T3 is to terminate spurious transcripts that might come from within the element, analogous to the proposed function of the terminators within the conjugation operon of the *B*. *subtilis* conjugative plasmid pLS20 [37].

After excision of Tn*916*, the promoter P*orf7* drives transcription of the DNA processing (nicking, unwinding, replication) and conjugation genes [19] and the terminators between P*orf7* and the end of the conjugation operon might be problematic for gene expression. However, the *canT* antiterminator RNA allows transcription to read through element terminators processively, enabling expression of DNA processing and conjugation genes essential for conjugation once the element is in the circularized form (Fig 1C).

This type of termination-antitermination system appears to be widespread in the large family of Tn*916*-like elements. We identified predicted intrinsic transcription terminators on the left end of several Tn*916*-like elements, including Tn*2010*, Tn*5251*, Tn*5386*, Tn*5397*, Tn*5801*, Tn*6000*, Tn*6002*, Tn6003, Tn*6084*, and Tn*6085a* (S6 Fig). Some of these terminators have identical sequences to the Tn*916* terminator T1, others have a few variations (S6A Fig), and some have completely different sequences (S6B Fig), but are predicted terminators nonetheless. In elements with terminators identical to Tn*916* terminator T1, the 452 bp *canT* sequence is also present. Some of the elements whose terminator efficiencies are not known have *canT*-like sequences at the right end and others lack any sequences that are easily recognized as resembling *canT*. We have not evaluated predicted RNA structures of transcriptions from these regions.

### Factors involved in processive transcription antitermination

**Fig 7:**
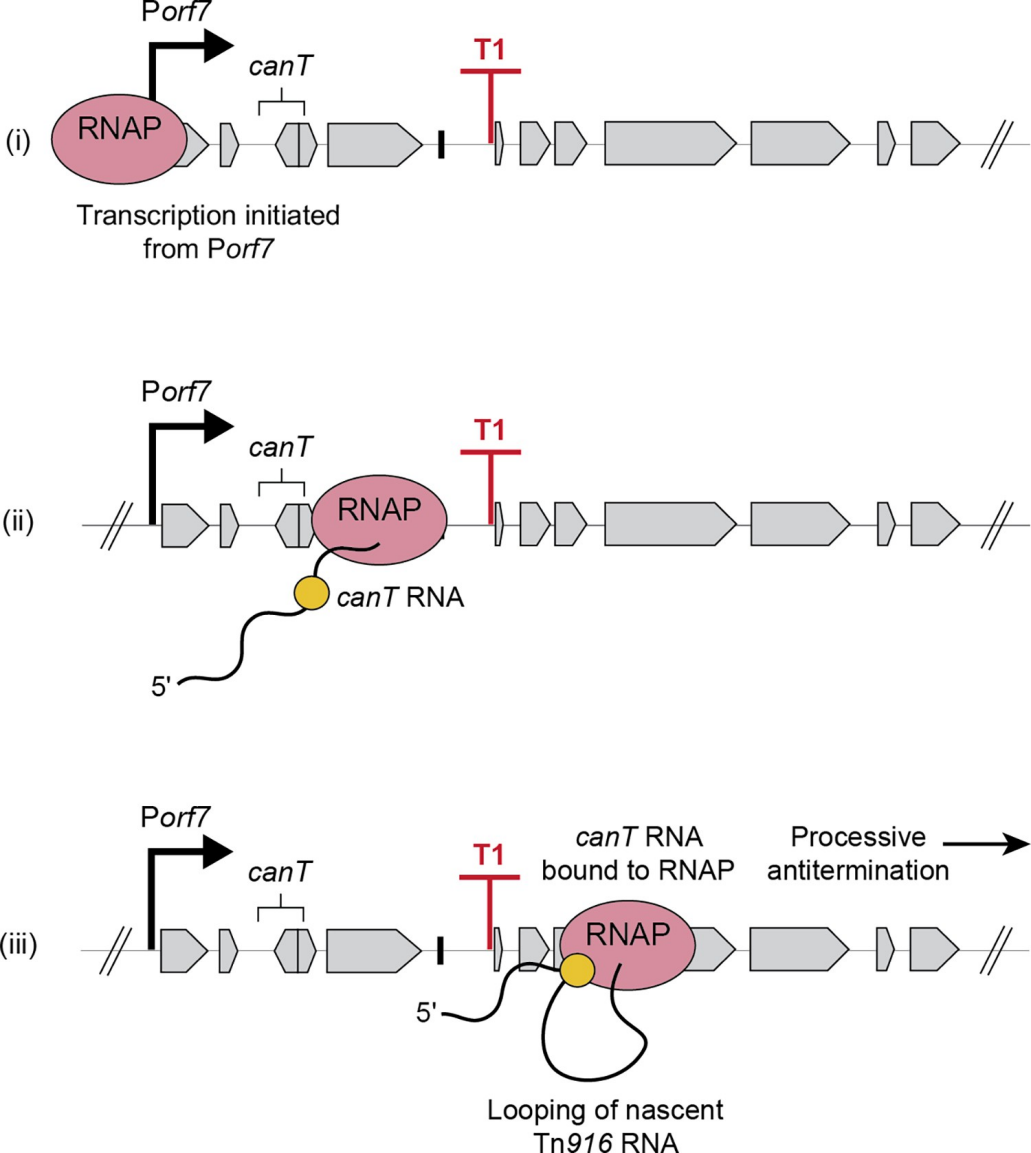
Cartoon for *canT*-mediated antitermination. (i) Post-excision, transcription by the RNA polymerase (pink) initiates at P*orf7* in the Tn*916* circle. (ii) The nascent RNA (wavy line) contains *canT* RNA (yellow circle). (iii) *canT* RNA binds to the RNA polymerase, modifying it into a terminator-resistant form, allowing processive antitermination. *canT* RNA-bound, modified RNA polymerase transcribes and bypasses terminator T1.

Processive transcription antitermination can involve protein and-or RNA factors, either acting independently or in combination. For example, the N and Q antitermination proteins of phage lambda bind to an RNA site (*nut* site for N utilization) or a DNA site near a promoter (*qut* site for Q utilization), and associate with RNA polymerase to cause antitermination [38,39,47–50]. The conjugative plasmid pLS20 encodes both the protein ConAn1 and the RNA *conAn2* that are used in antitermination of the long conjugation operon [37]. Antitermination systems in the lambdoid phage HK022 [40,41,51,52] and the *B*. *subtilis eps* operon [36] use RNA elements, *put* and EAR, respectively, for antitermination, without protein factors, although the possibility of host factor involvement has not been eliminated in the latter. Despite their differences, these factors are known or suspected to alter RNA polymerase such that it is no longer susceptible to most terminators [38,39,50,52–56], and some appear to act over distances up to ~30,000 nucleotides [36,37,57,58]. We suspect that Tn*916 canT* RNA directly interacts with RNA polymerase to make it resistant to most terminators (Fig 7).

### Biological roles of processive transcription antitermination

Based on our findings and comparisons with other systems, Tn*916 canT* likely serves several roles in the biology of Tn*916*. The presence of *canT* allows complete transcription of the long DNA processing and conjugation operon, despite the presence of internal terminators (discussed above). It functions to couple element gene expression to excision as *canT* allows transcripts from P*orf7* to read through the transcription terminators, most notably T1, when the element is in the circular form after excision (Fig 1C). Further, the presence of T1 and its function as an insulator to prevent element gene expression when Tn*916* is integrated downstream of a host promoter, would prevent Tn*916* (in its current form) from functioning as a conjugative element without a mechanism of antitermination. In this way, *canT* enables Tn*916* to have such an insulator and still function.

In addition to *canT*, P*orf7* is critical for the function of Tn*916*. Both are needed to couple element gene expression to excision. The DNA processing and conjugation operon is not expressed when Tn*916* is integrated due to the physical separation of the genes from the main promoter P*orf7* [19] (Fig 1B). Following excision, *canT* allows the transcript initiated from P*orf7* to bypass the internal terminators, leading to full expression of the operon in the Tn*916* extrachromosomal circle (Fig 1C). The use of antitermination to regulate timing of gene expression also occurs in the lambda phage, in which the N and Q proteins control the switch from immediate-early to delayed-early gene expression [38,39,47,50,59] and early to late gene expression [38,39,48–50,60], respectively.

### Mechanisms to prevent autonomous replication of integrated mobile genetic elements

ICEs have different mechanisms for regulating DNA nicking, unwinding and rolling circle replication. These mechanisms function to both couple autonomous replication with excision, and to stop new rounds of replication prior to or concomitant with integration. As described here, Tn*916* separates the main promoter and antiterminator from genes needed for DNA nicking, unwinding, and replication and uses a terminator upstream from those genes to prevent chromosomal promoters from reading into the element. This type of mechanism is enabled by the antitermination system in Tn*916*.

In contrast to Tn*916*, ICE*Bs1* uses a transcriptional repressor that controls expression of the excisionase (*xis*) and the DNA nicking, unwinding, and replication genes [42,61,62]. In this way, the DNA processing genes are expressed only when the excisionase is expressed. Further, repression and depletion of the excisionase is needed for integration, and this happens when *xis* is repressed, along with the DNA processing genes. This mechanism is similar to that used by some temperate phage, including lambda [63–66].

Our findings with Tn*916* highlight the intricate layers of transcriptional regulation in ICEs. We suspect that other ICEs may have similar termination-antitermination mechanisms and that this type of regulation is more widespread and may have a broader role in horizontal gene transfer than previously thought.

## Methods

### Media and growth conditions

*B*. *subtilis* cells were grown shaking at 37°C in either LB medium or MOPS (morpholinepropanesulfonic acid)-buffered 1X S7_50_ defined minimal medium [67] containing 0.1% glutamate, required amino acids (40 μg/ml phenylalanine and 40 μg/ml tryptophan) and either glucose or arabinose (1% w/v) as a carbon source or on LB plates containing 1.5% agar. *Escherichia coli* cells were grown shaking at 37°C in LB medium for routine strain constructions. Where indicated, tetracycline (2.5 μg/ml) was added to Tn*916*-containing cells to increase gene expression and excision [19]. Antibiotics were otherwise used at the following concentrations: 5 μg/ml kanamycin, 10 μg/ml tetracycline, 100 μg/ml spectinomycin, 5 μg/ml chloramphenicol, and a combination of erythromycin at 0.5 μg/ml and lincomycin at 12.5 μg/ml to select for macrolide-lincosamide-streptogramin (*mls*) resistance. 5-bromo-4-chloro-3-indolyl β-D-galactopyranoside (X-gal) was used at a concentration of 120μg/ml.

Prior to sample collection for β-galactosidase assay, cells were grown as light lawns on 1.5% agar plates containing 1% w/v glucose, 0.1% w/v monopotassium glutamate, and 1X Spizizen’s salts [2 g/l (NH_4_)SO_4_, 14 g/l K_2_HPO_4_, 6 g/l KH_2_PO_4_, 1 g/l Na_3_-citrate·2H_2_O, and 0.2 g/l MgSO_4_·7H_2_O] [68]. Cells were resuspended from light lawns and grown at 37°C with shaking in 1X S7_50_ defined minimal glucose medium [67].

### Strains, alleles, and plasmids

*E*. *coli* strain AG1111 (MC1061 F’ *lacI*^q^ *lacZ*M15 Tn*10*) was used as a host for various plasmids. *B*. *subtilis* strains (Table 1), except BS49, were derived from JMA222, a derivative of JH642 (*trpC2 pheA1*) [69,70] that is cured of ICE*Bs1* [71]. *B*. *subtilis* strains were constructed by natural transformation [68] or conjugation as indicated. Key strains and newly reported alleles are summarized below.

*ΔcomK*::*kan* in ELC37 replaced most of the *comK* open reading frame from 47 bp upstream of *comK* to 19 bp upstream of its stop codon with the kanamycin resistance cassette (*kan*) from pGK67 [72]. The *kan* marker was fused with up- and downstream homology regions via isothermal assembly [73] and used for transformation.

**Table 1:** *B*. *subtilis* strains used.

Strain	Relevant genotype^a^ (reference)	Comments
BS49	*metB5 hisA1 thr-5 att*(*yufKL*)::Tn*916 att*(*ykuC-ykyB*)::Tn*916* ICE*Bs1*^+^ [13,86,87]	
CMJ253	ICE*Bs1*^0^ *att*(*yufKL*)::Tn*916* [77,81]	
ELC37	ICE*Bs1*^0^ Δ*comK*::*kan*	
JMA222	ICE*Bs1*-cured (ICE*Bs1*^0^) [71]	
ESW98	ICE*Bs1*^0^ *att*(*yufKL*)::Tn*916 canT*(*S3*)	
ESW104	ICE*Bs1*^0^ *att*(*yufKL*)::Tn*916 orf5*(*M1I*)	
ESW120	ICE*Bs1*^0^ *att*(*yufKL*)::Tn*916 canT*(Δ*162*)	
ESW179	ICE*Bs1*^0^ *att*(*yufKL*)::[(Pspank-Tn*916*(T1^*+*^)) *mls*]	
ESW247	ICE*Bs1*^0^ *att*(*yufKL*)::[(Pspank-Tn*916*(T1^−^)) *mls*]	
ESW252	ICE*Bs1*^0^ *amyE*::[(Pspank-EMPTY-*lacZ*) *spc*]	termination assay
ESW261	ICE*Bs1*^0^ *att*(*yufKL*)::Tn*916*-*lacZ*^b^	
ESW270	ICE*Bs1*^0^ *att*(*yufKL*)::[(P*immR*-(*immR*-*immA*)) (P*xis*-Tn*916*(T1^*+*^)) *mls*]^c^ *amyE*::[(Pxyl-*rapI*) *spc*]	P*xis*-Tn*916* viability assay
ESW271	ICE*Bs1*^0^ *att*(*yufKL*)::[(P*immR*-(*immR*-*immA*)) (P*xis*-Tn*916*(T1^−^)) *mls*]^c^ *amyE*::[(Pxyl-*rapI*) *spc*]	P*xis*-Tn*916* viability assay
ESW316	ICE*Bs1*^0^ *att*(*yufKL*)::[(P*immR*-(*immR*-*immA*)) (P*xis*-Tn*916*(T1^−^ Δ*orf20*)) *mls*]^c^ *amyE*::[(Pxyl-*rapI*) *spc*]	P*xis*-Tn*916* viability assay
ESW363	ICE*Bs1*^0^ *att*(*yufKL*)::[(P*immR*-(*immR*-*immA*)) (P*xis*-Tn*916*(T1^−^ Δ*orf23*-*22*)) *mls*]^c^ *amyE*::[(Pxyl-*rapI*) *spc*]	P*xis*-Tn*916* viability assay
ESW398	ICE*Bs1*^0^ *amyE*::[(Pspank-*canT*(WT)-T1^+^-*lacZ*) *spc*]	antitermination assay; *canT*(WT); T1
ESW407	ICE*Bs1*^0^ *amyE*::[(Pspank-*canT*(*S3*)-T1^+^-*lacZ*) *spc*]	antitermination assay; *canT*(*S3*); T1
ESW408	ICE*Bs1*^0^ *amyE*::[(Pspank-*canT*(Δ*162*)-T1^+^-*lacZ*) *spc*]	antitermination assay of *canT*(Δ*162*); T1
ESW414	ICE*Bs1*^0^ *att*(*yufKL*)::[(P*immR*-(*immR*-*immA*)) (P*xis*-Tn*916*(T1^−^ Δ*oriT*)) *mls*]^c^ *amyE*::[(Pxyl-*rapI*) *spc*]	P*xis*-Tn*916* viability assay
ESW420	ICE*Bs1*^0^ *att*(*yufKL*)::Tn*916 orf5*(A30*)	allele of putative *orf5*
ESW421	ICE*Bs1*^0^ *amyE*::[(Pspank-*canT*(WT)-*gatB*TT^+^-*lacZ*) *spc*]^d^	antitermination assay; *canT*(WT); *gatB* terminator
ESW422	ICE*Bs1*^0^ *amyE*::[(Pspank-*canT* (291 bp)-T1^+^-*lacZ*) *spc*]	antitermination assay; *canT* (fragment)
ESW423	ICE*Bs1*^0^ *amyE*::[(Pspank-*canT* (232 bp)-T1^+^-*lacZ*) *spc*]	antitermination assay; *canT* (fragment)
ESW436	ICE*Bs1*^0^ *amyE*::[(Pspank-*canT* (349 bp)-T1^+^-*lacZ*) *spc*]	antitermination assay; *canT* (fragment)
ESW437	ICE*Bs1*^0^ *amyE*::[(Pspank-*canT* (400 bp)-T1^+^-*lacZ*) *spc*]	antitermination assay; *canT* (fragment)
ESW438	ICE*Bs1*^0^ *amyE*::[(Pspank-*canT* (290 bp)-T1^+^-*lacZ*) *spc*]	antitermination assay; *canT* (fragment)
ESW440	ICE*Bs1*^0^ *amyE*::[(Pspank-*canT*(Flipped)-T1^+^-*lacZ*) *spc*]	antitermination assay; *canT* (flipped); T1
ESW449	ICE*Bs1*^0^ *amyE*::[(Pspank-*canT*(*S3*)- *gatB*TT ^+^-*lacZ*) *spc*]^d^	antitermination assay of *canT*(*S3*); *gatB* terminator
ESW450	ICE*Bs1*^0^ *amyE*::[(Pspank-*canT* (373 bp)-T1^+^-*lacZ*) *spc*]	antitermination assay; *canT* (fragment)
ESW459	ICE*Bs1*^0^ *amyE*::[(Pspank-*canT* (*S3*, 400 bp)-T1^+^-*lacZ*) *spc*]	antitermination assay; *canT*(*S3*) (fragment)
ESW473	ICE*Bs1*^0^ *amyE*::[(Pspank-*canT*(Δ*162*)- *gatB*TT ^+^-*lacZ*) *spc*]^d^	antitermination assay; *canT*(Δ*162*); *gatB* terminator
ESW481	ICE*Bs1*^0^ *amyE*::[(Pspank-*canT*(Δ*162*)-T2^+^-*lacZ*) *spc*]	antitermination assay; *canT*(Δ*162*); T2
ESW482	ICE*Bs1*^0^ *amyE*::[(Pspank-*canT*(Δ*162*)-T3^+^-*lacZ*) *spc*]	antitermination assay; *canT*(Δ*162*); T3
ESW492	ICE*Bs1*^0^ *amyE*::[(Pspank-*canT*(WT)-T2^+^-*lacZ*) *spc*]	antitermination assay; *canT*(WT); T2
ESW493	ICE*Bs1*^0^ *amyE*::[(Pspank-*canT*(WT)-T3^+^-*lacZ*) *spc*]	antitermination assay; *canT*(WT); T3
ESW517	ICE*Bs1*^0^ *att*(*sdpA*)::Tn*916*-*lacZ*	Tn*916-lacZ* insertion
ESW545	ICE*Bs1*^0^ *amyE*::[(Pspank-*canT* (WT, 341 bp)-T1^+^-*lacZ*) *spc*]	antitermination assay; *canT* (fragment)
ESW557	ICE*Bs1*^0^ *att*(*fadR*)::Tn*916*-*lacZ*	Tn*916-lacZ* insertion
ESW561	ICE*Bs1*^0^ *att*(*adaB*-*ndhF*)::Tn*916*-*lacZ*	Tn*916-lacZ* insertion
ESW562	ICE*Bs1*^0^ *att*(*sunT*)::Tn*916*-*lacZ*	Tn*916-lacZ* insertion
ESW605	ICE*Bs1*^0^ *amyE*::[(Pspank-T1^+^-*lacZ*) *spc*]	termination assay; T1
ESW606	ICE*Bs1*^0^ *amyE*::[(Pspank-T1^−^-*lacZ*) *spc*]	termination assay; T1^−^
ESW616	ICE*Bs1*^0^ *amyE*::[(EMPTY) *spc*]	pairwise competition
ESW617	ICE*Bs1*^0^ *amyE*::[(Ppen-*lacZ*) *spc*]	pairwise competition
ESW623	ICE*Bs1*^0^ *amyE*::[(Pspank-*canT*(*S3*)-T2^+^-*lacZ*) *spc*]	antitermination assay; *canT*(*S3*); T2
ESW626	ICE*Bs1*^0^ *amyE*::[(Pspank-*canT*(*S3*)-T3^+^-*lacZ*) *spc*]	antitermination assay; *canT*(*S3*); T3
ESW630	ICE*Bs1*^0^ *att*(*yufKL*)::Tn*916* T1^–^ *canT*(*S3*)	
ESW675	ICE*Bs1*^0^ *att*(*sdpA*)::Tn*916* T1^+^ *amyE*::[(Ppen-*lacZ*) *spc*]	pairwise competition
ESW677	ICE*Bs1*^0^ *att*(*sunT*)::Tn*916* T1^+^ *amyE*::[(Ppen-*lacZ*) *spc*]	pairwise competition
ESW693	ICE*Bs1*^0^ *att*(*sdpA*)::Tn*916* T1^–^ *amyE*::[(EMPTY) *spc*]	pairwise competition
ESW695	ICE*Bs1*^0^ *att*(*sunT*)::Tn*916* T1^–^ *amyE*::[(EMPTY) *spc*]	pairwise competition
ESW713	ICE*Bs1*^0^ *att*(*yvgT*-*bdbC*)::Tn*916* T1^+^ *amyE*::[(Ppen-*lacZ*) *spc*]	pairwise competition
ESW714	ICE*Bs1*^0^ *att*(*yvgT*-*bdbC*)::Tn*916* T1^–^ *amyE*::[(EMPTY) *spc*]	pairwise competition
ESW722	ICE*Bs1*^0^ *att*(*adaB-ndhF*)::Tn*916* T1^+^ *amyE*::[(Ppen-*lacZ*) *spc*]	pairwise competition
ESW723	ICE*Bs1*^0^ *att*(*adaB-ndhF*)::Tn*916* T1^–^ *amyE*::[(EMPTY) *spc*]	pairwise competition
ESW724	ICE*Bs1*^0^ *att*(*fadR*)::Tn*916* T1^+^ *amyE*::[(Ppen-*lacZ*) *spc*]	pairwise competition
ESW725	ICE*Bs1*^0^ *att*(*fadR*)::Tn*916* T1^–^ *amyE*::[(EMPTY) *spc*]	pairwise competition
ESW728	ICE*Bs1*^0^ *att*(*adaB-ndhF*)::Tn*916* Δ*oriT* T1^–^ *amyE*::[(EMPTY) *spc*]	pairwise competition
ESW771	ICE*Bs1*^0^ *att*(*yufKL*)::Tn*916 canT*(*S3*) *amyE*::[(Pspank-*canT*(WT)-T1^+^-*lacZ*) *spc*]	*canT* functions in cis
ESW781	ICE*Bs1*^0^ *att*(*yufKL*)::Tn*916 amyE*::[(Pspank-*canT*(WT)-T1^+^-*lacZ*) *spc*]	*canT* functions in cis
ESW783	ICE*Bs1*^0^ *amyE*::[(Pspank-*canT*(WT)-*rrnB* T1^+^, *rrnB* T2^+^, λ t0^+^-*lacZ*) *kan*]	antitermination assay; *canT*(WT); 3 terminators
ESW784	ICE*Bs1*^0^ *amyE*::[(Pspank-*canT*(*S3*)-*rrnB* T1^+^, *rrnB* T2^+^, λ t0^+^-*lacZ*) *kan*]	antitermination assay; *canT*(*S3*); 3 terminators
ESW785	ICE*Bs1*^0^ *amyE*::[(Pspank-*canT*(Δ*162*)-*rrnB* T1^+^, *rrnB* T2^+^, λ t0^+^-*lacZ*) *kan*]	antitermination assay; *canT*(Δ*162*); 3 terminators

^a^All strains, except BS49, are derived from JH642 and contain the *trpC2 pheA1* alleles [69,70]. These alleles are not indicated in the table. Strains do not contain Tn*916* unless Tn*916* is specifically indicated.

^b^Tn*916*-*lacZ* contains *lacZ* between *attL* and *orf24*.

^c^P*xis*-driven alleles use the P*xis* promoter from ICE*Bs1*, which is repressed by ImmR and activated by the metalloprotease ImmA and the cell-signaling receptor RapI [42,61,71].

^d^*gatB*TT^+^ indicates wild-type sequence of a terminator located downstream of *gatB* gene in *B*. *subtilis* genome.

#### Unmarked deletions and mutations in Tn*916*

Unmarked deletions and point mutations in Tn*916* were generated by a two-step allelic replacement approach. Briefly, DNA flanking the regions to be altered were amplified and inserted by isothermal assembly [73] into the *EcoRI* and *BamHI* sites of pCAL1422, a plasmid containing *E*. *coli lacZ* and *B*. *subtilis* chloramphenicol resistance cassette (*cat*), as previously described [45,74]. For deletion mutations, there was no DNA between the flanking regions. For point mutations, a fragment containing the desired mutations was assembled between the flanking regions. The resulting plasmids were used to transform the appropriate *B*. *subtilis* strains, selecting for integration of the plasmid into the chromosome (chloramphenicol-resistant) by single crossover recombination. Transformants were screened for loss of *lacZ* and checked by PCR and sequencing for the desired allele. Deletions and point mutations are described below.

- *Genes and sites involved in replication*. Deletion of genes *orf23*-*22* (encoding helicase processivity factors) extends from immediately after the stop codon of *orf24* through the stop codon of *orf22*. Deletion of *orf20* (encoding the relaxase) fuses the first 90 codons of *orf20* with the *orf20* stop codon, deleting the intervening 306 codons and preserving *oriT* [75]. Deletion of *oriT* (Δ*oriT*) removes 5’- CCCCCCGTAT CTAACAGGGG GG-3’, starting from the nucleotide 41 downstream from the stop codon of *orf21*.
- *Mutations in the putative* orf5. *orf5*(*M1I*) (ESW104) changes the predicted start codon from AUG to AUA. This mutation preserves the amino acid sequence of the overlapping *xis*. *orf5*(A30*) (ESW420) changes codon 30 (of 83 total) from GCA (alanine) to UAA (stop).
- *Mutations in* canT. Deletion of *canT* [*canT*(*Δ162*), ESW120] removes 162 bp between *orf8* and *xis*, extending from 229 to 390 nucleotides downstream from the stop codon of *orf8* (Fig 3B). *canT*(*S3*) (ESW98) contains a 3 bp substitution 5’-ATA-3’ to 5’-TGC-3’, starting from the nucleotide 283 downstream from the stop codon of *orf8* (Fig 3B).
- *Mutations in the terminator T1*. Multiple base pair changes were made to inactivate terminator T1 (T1^–^), including changing three nucleotides in the predicted stem of the stem-loop, two nucleotides near the base of the stem, and two of the U’s that follow the stem in the predicted RNA secondary structure (Fig 1D). The mutations did not alter the ribosome binding site or amino acid sequence of *orf24*, which overlaps T1.

#### Pspank-Tn*916* and P*xis*-Tn*916*

Pspank-Tn*916* strains (T1^+^, ESW179; T1^–^, ESW247) and P*xis*-Tn*916* strains (T1^+^, ESW270; T1^–^, ESW271; T1^–^ Δ*orf20*, ESW316; T1^–^ Δ*orf23-22*, ESW363; T1^–^ Δ*oriT*, ESW414) were created by cloning, either Pspank or ICE*Bs1* P*xis* promoter, and an MLS resistance cassette (*mls*) upstream of *att*(*yufKL*)::Tn*916* and its derivative mutants. DNA flanking the regions to be altered were generated by PCR and assembled flanking the promoter and *mls* via isothermal assembly [73]. DNA was transformed into *B*. *subtilis* selecting for resistance to MLS. Transcription from P*xis* was derepressed by overexpression of *rapI* under the control of a xylose-inducible promoter inserted at the non-essential locus *amyE* [*amyE*::(Pxyl-*rapI*) *spc*] [44]. RapI causes the protease ImmA to cleave ImmR, the repressor of P*xis*, thereby derepressing transcription from P*xis* [42,43].

#### Tn*916-lacZ*

Tn*916*-*lacZ* (ESW261) has *lacZ* at the left end of Tn*916*, upstream of T1 (Fig 6A) and was used to identify insertions that were downstream from an active promoter. Briefly, *lacZ* and a kanamycin resistance gene (*kan*) that was flanked by *lox* sites were inserted 29 bp upstream of *orf24* (upstream of T1) by isothermal assembly [73] and introduced into Tn*916*. The Cre recombinase, expressed from the temperature-sensitive plasmid, pDR244 [76], was then used to remove the *lox*-flanked *kan* marker by recombination. Strains were then cured of pDR244 by culturing them on LB agar at 42°C, as previously described [76,77]. *lacZ* is expressed when integrated Tn*916* is integrated downstream from an active promoter.

#### *lacZ* reporter for measuring transcription termination and antitermination

DNA fragments with possible terminators upstream from *lacZ* were cloned downstream of Pspank in the vector pDR110 (a gift from D. Rudner, integrates by double crossover at *amyE*; contains Pspank, *lacI*, *spc*). Briefly, *lacZ* was amplified by PCR from pCAL1422 [45] using primers that include either wild-type or mutant T1. The T1 sequence cloned included 6 bp and 15 bp directly upstream and downstream, respectively, of the base of the stem of the terminator hairpin. The PCR products were inserted by isothermal assembly [73] into pDR110, cut with *NheI* and *HindIII* and the resulting construct was integrated into *B*. *subtilis* at the *amyE* locus by recombination selecting for resistance to spectinomycin. Reporter strains contained: no terminator (ESW252); T1^+^ (ESW605); and the T1 mutant (T1^–^, ESW606).

Antitermination activity was measured using a reporter with a terminator between Pspank and *lacZ* and cloning additional DNA fragments containing the indicated *canT* alleles between Pspank and the indicated terminator. The 452 bp DNA fragment with wild-type *canT* extends from 126 to 577 bp downstream of the stop codon of *orf8* (Fig 3B). The strategy for building each construct was similar to that described for the terminators above. Strains contained the *canT* alleles: WT, Δ*162*, *S3*, or flipped, followed by T1 (ESW398, ESW408, ESW407, ESW440, respectively); the WT, Δ*162*, *S3* alleles upstream of T2 (ESW492, ESW481, ESW623, respectively); upstream of T3, (ESW493, ESW482, ESW626, respectively); or upstream of the *gatB* terminator (ESW421, ESW473, ESW449).

Additionally, each of the three different *canT* alleles (WT, Δ*162*, *S3*) was cloned upstream from an array of three terminators, *E*. *coli rrnB* T1, *rrnB* T2, and the phage lambda λ T0, between Pspank and *lacZ* and integrated into the chromosome at *amyE*, essentially as described above, generating strains ESW783 (WT), ESW785 (Δ*162*), and ESW784 (*S3*).

### β-galactosidase assays

Cells were grown at 37°C in defined minimal medium with shaking. At OD_600_ ~0.1, IPTG was added (1 mM final concentration) to induce transcription from Pspank and samples were taken two hours post-induction. In other experiments, cells were grown on LB agar plates and harvested after the indicated time of growth. Cells were permeabilized with 15 μl of toluene and β-galactosidase specific activity was determined [(ΔA420 per min per ml of culture per OD_600_ unit) × 1000] essentially as described [78] after pelleting cell debris.

### RT-qPCR to measure gene expression

For reverse transcription reactions, an aliquot of cells was harvested in ice-cold methanol (1:1 ratio) and pelleted. RNA was isolated using Qiagen RNeasy PLUS kit with 10 mg/ml lysozyme. iScript Supermix (Bio-Rad) was used for reverse transcriptase reactions to generate cDNA. Control reactions without reverse transcriptase were performed to assess the amount of DNA present in the RNA samples. RNA was degraded by adding 75% volume of 0.1 M NaOH, incubating at 70°C for 10 min, and neutralizing with an equal volume of 0.1 M HCl.

The relative amounts of cDNA were determined by qPCR using SSoAdvanced SYBR master mix and CFX96 Touch Real-Time PCR system (Bio-Rad). qPCR data were quantified using the standard curve method [79]. Standard curves for these reactions were generated using *B*. *subtilis* genomic DNA that contained wild-type Tn*916*. Primers used to quantify *xis*, *orf23*, *orf22*, *orf21*, *orf20*, and the chromosomal locus *gyrA* are listed in Table 2. The relative mRNA levels of Tn*916* genes (as indicated by the Cq values measured by qPCR) were normalized to *gyrA* after subtracting the signal from control reaction without reverse transcriptase.

**Table 2:** Primers used for qPCR and RT-qPCR.

Gene/region	Primer (reference)	Sequence (5’ to 3’)	Comments
*xis*	oESW48	CTAACCATTG AAGAAGCGTC AAA	RT-qPCR
	oESW49	ACGATTGCCA TTCATAATCA GC	RT-qPCR
*orf23*	oELC436	GAAATGTTTT TCGCCAGCTT CAGC	RT-qPCR
	oELC437	GCGAAGAATC AACGGACGGC	RT-qPCR
*orf22*	oLW443	CTCTACGTCG TGAAGTGAGA ATCC	RT-qPCR
	oLW444	TTGATAAGTT CCACCCGTGC G	RT-qPCR
*orf21*	oELC438	CCTCACTACG TTTCATCATT TCTTCATAGA ATG	RT-qPCR
	oELC439	GCTGACCTTG CGGACTTAGG	RT-qPCR
*orf20*	oELC440	CTCTTTGCGT ACCAGTTCGC C	RT-qPCR
	oELC441	GACCTTGCCA TTAACGATAA GACAGG	RT-qPCR
*gyrA*	oMEA128	TGGAGCATTACCTTGACCATC	RT-qPCR
	oMEA129	AGCTCTCGCTTCTGCTTTAC	RT-qPCR
Tn*916* empty chromosomal attachment site (*att1*)	oLW542 [18]	GCAATGCGAT TAATACAACG ATAC	qPCR
	oLW543 [18]	TCGAGCATTC CATCATACAT TC	qPCR
Tn*916* circular junction (*att*Tn*916*)	oLW526 [18]	AAACGTGAAG TATCTTCCTA CAG	qPCR
	oLW527 [18]	TCGTCGTATC AAAGCTCATT C	qPCR
*mrpG*	oLW544 [18]	CCTGCTTGGG ATTCTCTTTA TC	qPCR
	oLW545 [18]	GTCATCTTGC ACACTTCTCT C	qPCR

### qPCR to measure Tn*916* excision and circle copy number

qPCR was used to monitor excision (activation) and copy number of circular (excised) Tn*916*, essentially as described previously [18,80,81]. Briefly, cells containing Tn*916* were lysed (40 mg/ml lysozyme) and genomic DNA was prepared using the Qiagen DNeasy kit. qPCR was performed using SsoAdvanced SYBR master mix and the CFX96 Touch Real-Time PCR system (Bio-Rad). qPCR data were quantified using the Pfaffl method [82]. Standard curves for these qPCRs were generated using *B*. *subtilis* genomic DNA that contained an empty Tn*916* chromosomal attachment site (*att1*), an ectopic copy of the Tn*916* circle *att*Tn*916* junction inserted at *amyE*, and a copy of the nearby locus, *mrpG*.

Excision frequencies were calculated as the number of copies of the chromosomal site from which Tn*916* excised (*att1*) divided by the number of copies of *mrpG* (a nearby gene). The average number of copies of the circular Tn*916* per cell was calculated as the number of copies of *att*Tn*916* divided by the number of copies of *mrpG*. Primers used to amplify the empty chromosomal attachment site (*att1*), the *att*Tn*916* junction in the circular Tn*916*, and a region within the nearby gene *mrpG* were described previously [18] and are listed on Table 2.

### Growth and viability assays

P*xis*-Tn*916* strains were grown in defined minimal medium with 1% arabinose as a carbon source to early exponential phase. At an OD_600_ of 0.05, the cultures were split and xylose was added (1% final concentration) to one portion to induce transcription from Pxyl, thus expressing *rapI* (Pxyl-*rapI*), causing inactivation of ImmR, the repressor of P*xis* [42,43]. After four hours, the number of CFUs was determined in induced and non-induced cultures. “Relative viability” was calculated as the number of CFUs present in the induced culture divided by the number of CFUs present in the non-induced culture.

### Mating assay

Mating assays were performed essentially as described previously [71]. Briefly, donor strains containing Tn*916* (tetracycline-resistant) or derivatives were grown in LB medium to early exponential phase. At an OD_600_ ~0.2, activation of Tn*916* was stimulated by addition of tetracycline (2.5 μg/ml final concentration). After one hour, donor strains were mixed in a 1:1 ratio with kanamycin-resistant recipient cells (ELC37) and 5 total OD units of cells were filtered. Mating filters were placed on a 1X Spizizen’s salts [68] 1.5% agar plate at 37°C for one hour. Cells were then harvested off the filter and the number of CFUs of donors (tetracycline-resistant), recipients ELC37 (kanamycin-resistant), and transconjugants (tetracycline/kanamycin-resistant) were determined both pre- and post-mating. Conjugation efficiency is the percentage of transconjugants per donor (using the number of donors determined at the start of mating).

### Identification of Tn*916* insertions downstream from active host promoters

We used Tn*916-lacZ* to identify insertions downstream from host promoters. A donor containing Tn*916-lacZ* (ESW261) was crossed to a recipient without Tn*916* (ELC37) and cells were plated on LB agar containing tetracycline to select for transconjugants, kanamycin to kill donors (counterselection), and X-gal to screen for insertions downstream from an active host promoter, as indicated by blue colony color. Approximately 10–15% of the initial transconjugants appeared blue after overnight growth on selective LB agar plates. Blue transconjugant colonies were picked and re-streaked non-selectively on LB agar containing X-gal. After re-streaking, many of the initially light blue colonies appeared white, indicating that there was little or no expression of *lacZ*. We suspect that the initial light blue appearance was from *lacZ* expression in the nascent transconjugant, before the element had integrated. We subsequently confirmed that candidates of interest were resistant to tetracycline (encoded by Tn*916-lacZ*).

### Mapping Tn*916*-*lacZ* integration sites

Arbitrary PCR was used to map Tn*916*-*lacZ* integration sites, as previously described [83,84]. Briefly, blue, tetracycline-resistant colonies were used as a template in a PCR reaction containing arbitrary primers (oELC1003: 5’- GGCACGCGTC GACTAGTACN NNNNNNNNNT GATG-3’) paired with a primer to either the right (oELC1009: 5’- GACATGCTAA TATAGCCATG ACG-3’) or left (oELC1010: 5’-GAAGTATCTT TATATCTTCA CTTTTCAAGG-3’) end of Tn*916*. Purified PCR products were then amplified using oELC1004 (5’-GGCACGCGTC GACTAGTAC-3’) and oELC1011 (5’-GAACTATTAC GCACATGCAA C-3’) for the right junction or oELC1012 (5’-CGTCGTATCA AAGCTCATTC ATAAG-3’) for the left junction. These PCR products were then sequenced with oELC1011 or oELC1012 and mapped to *B*. *subtilis* genome (Genbank accession number CP007800 [70]).

### Competition assays and fitness

#### Strains and growth

Tn*916* T1^+^ and T1^–^ strains were created by first replacing *lacZ* from Tn*916*-*lacZ* with *kan* flanked by *lox* sites and then removing *kan*. DNA fragments from upstream and downstream of *lacZ* (in Tn*916-lacZ*) were assembled by isothermal assembly [73] with either T1^+^ or T1^–^ and the *lox-*flanked *kan* and recombined into each Tn*916-lacZ* insertion by transformation and selection for resistance to kanamycin. The *lox*-flanked *kan* marker was then removed by Cre-mediated recombination (using pDR244 [76] as described above). Strains made include Tn*916* inserted in: *sdpA* (T1^+^, ESW675; T1^–^, ESW693), *sunT* (T1^+^, ESW677; T1^–^, ESW695), *fadR* (T1^+^, ESW724; T1^–^, ESW725), *adaB-ndhF* (T1^+^, ESW722; T1^–^, ESW723; T1^–^ Δ*oriT*, ESW728), *yvgT-bdbC* (T1^+^, ESW713; T1^–^, ESW714).

Because the insertions no longer contained *lacZ*, we were able to use a constitutively expressed *lacZ* (Ppen-*lacZ*) at *amyE* in the Tn*916* T1^+^ strains to distinguish them from the T1^–^ strains. *amyE*::[(Ppen-*lacZ*), *spc*] (ESW617) was made by PCR amplifying Ppen-*lacZ* from pCAL1422 [45] and inserting it into *BamHI*-*BlpI*-cut pAJW82 (a pDR110-derived vector that is lacking Pspank and *lacI*) by isothermal assembly [73], and then integrating it into *B*. *subtilis*. The cells with Tn*916* T1^–^ contained *amyE*::*spc* (ESW616) from pAJW82 with no insert. Competition experiments demonstrated that Ppen-*lacZ* did not affect fitness.

Strains with Tn*916* containing a wild-type or mutant T1 were grown in LB medium to early exponential phase. Strains were then mixed at the indicated ratio after adjusting their OD_600_ to ~0.01 and 50 μl of each competition mixture was spotted on LB agar and grown for 24 hours (1 day) at 37°C. Every 24 hours, spots were resuspended in 1X Spizizen’s salts [68] and diluted to an OD_600_ of ~0.01 and then 50 μl of this resuspension was spotted on fresh LB agar and grown for another 24 hours at 37°C. This was repeated for a total of 5 days. Under these conditions, cells progress through ~10 doublings per growth cycle on LB agar. At generation (doubling) 0 (initial input) and every 24 hours, the ratio of the two strains in each mixture was determined by serially diluting and plating the appropriate dilution on LB agar containing X-gal and counting the number of *lacZ*^+^ (blue; Tn*916* T1^+^) and *lacZ*^−^(white; Tn*916* T1^–^) colonies.

#### Fitness calculations

We calculated the fitness of the strain containing the mutant relative to that of the strain containing the wild-type terminator T1 using the equation derived from the Malthusian parameter estimate of fitness as described [85].

$$w_{T1mut}=\frac{\ln\left(\frac{N_{T1mut}(f)\times d}{N_{T1mut}(i)}\right)}{\ln\left(\frac{N_{WT}(f)\times d}{N_{WT}(i)}\right)}$$

*w*_*T*1*mut*_ is the fitness of the strain with Tn*916* T1^–^ relative to that with Tn*916* T1^+^. *N*_*T*1*mut*_ and *N_WT_* are the numbers of CFU/ml of the strains with the mutant and wild-type T1, respectively. *i* indicates the initial number of CFU/ml of the indicated strain in the mixture and *f* indicates the CFU/ml after growth of the mixed strains. *d*, the dilution factor, is the fold-dilution of the cells from the start of the experiment (*i*) to the time (*f*) at which fitness was determined. Fitness was determined at a time when the population size was still changing (Fig 6D–6J). The dilution factor used to calculate fitness was ~1,000 (two days of growth) for strains with insertions in *sdpA*, *sunT*, *adaB-ndhF* Δ*oriT*, and *yvgT-bdbC*, and the strains used for the *lacZ* control, and 1 (only one day of growth) for strains with insertions in *fadR* and *adaB-ndhF*.

Control competitions were performed to determine the fitness associated with the *amyE*::[(EMPTY) *spc*] marker used in Tn*916* T1^–^ cells (ESW616) relative to the *amyE*::[(Ppen-*lacZ*) *spc*] marker (ESW617) used in Tn*916* T1^+^ cells. The relative fitness was 1.02 ± 0.02 (mean ± SEM from three independent experiments), indicating that *lacZ* expression did not affect fitness of host cells (Fig 6C).

## Supporting information

S1 Fig Terminator T2 and T3 of Tn*916*. The nucleotide positions of the base of the terminator stem were determined by the ARNold web server [28,29]. The minimum free energy of folding ΔG (of the stem-loop) was calculated using the RNAfold web server [31]. **A**) Terminator T2a and T2b. Red, bolded UAA indicate the stop codon of *orf18*. **B)** Terminator T3.
(TIF)

S2 Fig Pspank-Tn*916* cannot excise and conjugate. **A)** Number of circular Tn*916* per cell (*att*Tn*916*/*mrpG*) and **B)** conjugation efficiencies of wild-type Tn*916* (CMJ253), Pspank-Tn*916* T1^+^ (ESW179), and Pspank-Tn*916* T1^–^ (ESW247). All strains were grown without tetracycline. Pspank-Tn*916* strains were grown continuously with IPTG. Data presented are from one experiment. Mating assays of Pspank-Tn*916* T1^+^ and Pspank-Tn*916* T1^–^ resulted in conjugation efficiencies that are below the limit of detection.
(TIF)

S3 Fig Analysis of the antitermination activity of different DNA fragments from the *canT* region of Tn*916*. **A)** Schematic of *lacZ* reporter construct with *canT* alleles and terminator T1 between Pspank and *lacZ*. **B-I)** DNA regions tested for antitermination activity. The size of each fragment is shown. The effect caused by *canT*(*S3*) was determined for two of the cloned fragments (**B,C**). β-galactosidase specific activities were measured two hours after induction of Pspank with IPTG and relative specific activities were calculated as the mean β-galactosidase specific activity of each strain divided by the mean β-galactosidase specific activity of the strain with the 452 bp fragment of the wild type *canT* allele. **B)** [*canT* (WT, 452 bp), ESW398] and [*canT* (*S3*, 452bp), ESW407]. Data for each are from three independent experiments. **C)** [*canT* (WT, 400 bp), ESW437] and [*canT* (*S3*, 400bp), ESW459]. Data for each are from three independent experiments. **D)** [*canT* (WT, 373 bp), ESW450]. Data presented are from three independent experiments. **E)** [*canT* (WT, 349 bp), ESW436]. Data presented are from one experiment. **F)** [*canT* (WT, 291 bp), ESW422]. Data presented are from one experiment. **G)** [*canT* (WT, 341 bp), ESW545]. Data presented are from three independent experiments. **H)** [*canT* (WT, 290 bp), ESW438]. Data presented are from one experiment. **I)** [*canT* (WT, 232 bp), ESW423]. Data presented are from one experiment.
(TIF)

S4 Fig Predicted secondary structures of different RNAs from the *canT* region of Tn*916*. RNA secondary structure predictions were generated using the ViennaRNA package [30] in the SnapGene software. Secondary structures shown are calculated to have the lowest predicted free energy. 5’ and 3’ ends of the RNA are indicated. The predicted structures are colored based on estimated confidence on bases being paired or unpaired (red: 90% and greater, yellow: 70–89%, light blue: 50–69%, dark blue: less than 50%). Structures within green and blue boxes are referred to as the left and right sides of the predicted structures. **A) Predicted secondary structure of the 452-nucleotide wild-type *canT* RNA.** The location of *S3* mutation is indicated (although the structure is that predicted for the wild-type). **B) Predicted secondary structure of the mutant *canT*(Δ*162*) RNA.** The 5’ and 3’ boundaries are the same as those for the 452-nucleotide wild-type *canT* RNA, but with 162 nucleotides deleted. See Fig 3B for details on the deletion. This allele is inactive in antitermination. **C) Predicted secondary structure of the 452-nucleotide mutant *canT*(*S3*) RNA.** The *canT*(*S3*) mutation changes 3 nucleotides (5’-AUA-3’ to 5’-UGC-3’) as shown. This allele is inactive in antitermination. **D) Predicted secondary structure of a 291-nucleotide RNA fragment from the *canT* region.** This allele is inactive in antitermination. **E) Predicted secondary structure of a 341 nucleotide RNA fragment from the *canT* region.** This fragment has antitermination activity.
(TIF)

S5 Fig Expression of host promoters upstream of four Tn*916* insertions. The original Tn*916*-*lacZ* insertions were used to monitor expression from host promoters under the conditions indicated. In both panel A and B, data presented are from one experiment. **A)** β-galactosidase activities are plotted as a function of cell density (OD_600_) during growth in defined liquid glucose medium at 37°C. The slope of each line is the differential rate of synthesis from the indicated promoter. Circles, *sdpA*::Tn*916*-*lacZ* (ESW517); squares, *sunT*::Tn*916*-*lacZ* (ESW562); triangles, *fadR*::Tn*916*-*lacZ* (ESW557); inverted triangles, *adaB-ndhF*::Tn*916*-*lacZ* (ESW561). **B)** β-galactosidase specific activities of Tn*916*-*lacZ* insertion strains grown as spots on LB agar at 37°C. Measurements were taken from spots resuspended in buffer after 8 hours of growth. Strains used were the same as in panel A.
(TIF)

S6 Fig DNA sequence for putative transcription terminators near the left end of Tn*916*-like elements. Yellow-highlighted sequences are the predicted stem-loops of the putative terminators near the left end of the indicated elements. None of these have been tested experimentally for terminator activity. **A)** Sequences that are identical to that of the Tn*916* terminator T1 or have a few nucleotide differences in the loop or regions upstream and downstream from the predicted stems. **B)** Tn*5386*, Tn*5397*, Tn*5801* have predicted terminators with different sequences than that of Tn*916* terminator T1.
(TIF)

S1 Data Underlying raw data for experiments presented in the figures. The excel spreadsheet contains the underlying data for the experiments presented in each of the figures.
(XLSX)
