# Supplementary figures and images for: Transcription termination and antitermination are critical for the fitness and function of the integrative and conjugative element Tn916
Source: PLoS Genet. 2024 Dec 9;20(12):e1011417. doi: 10.1371/journal.pgen.1011417 (PMC11658703; doi:10.1371/journal.pgen.1011417)

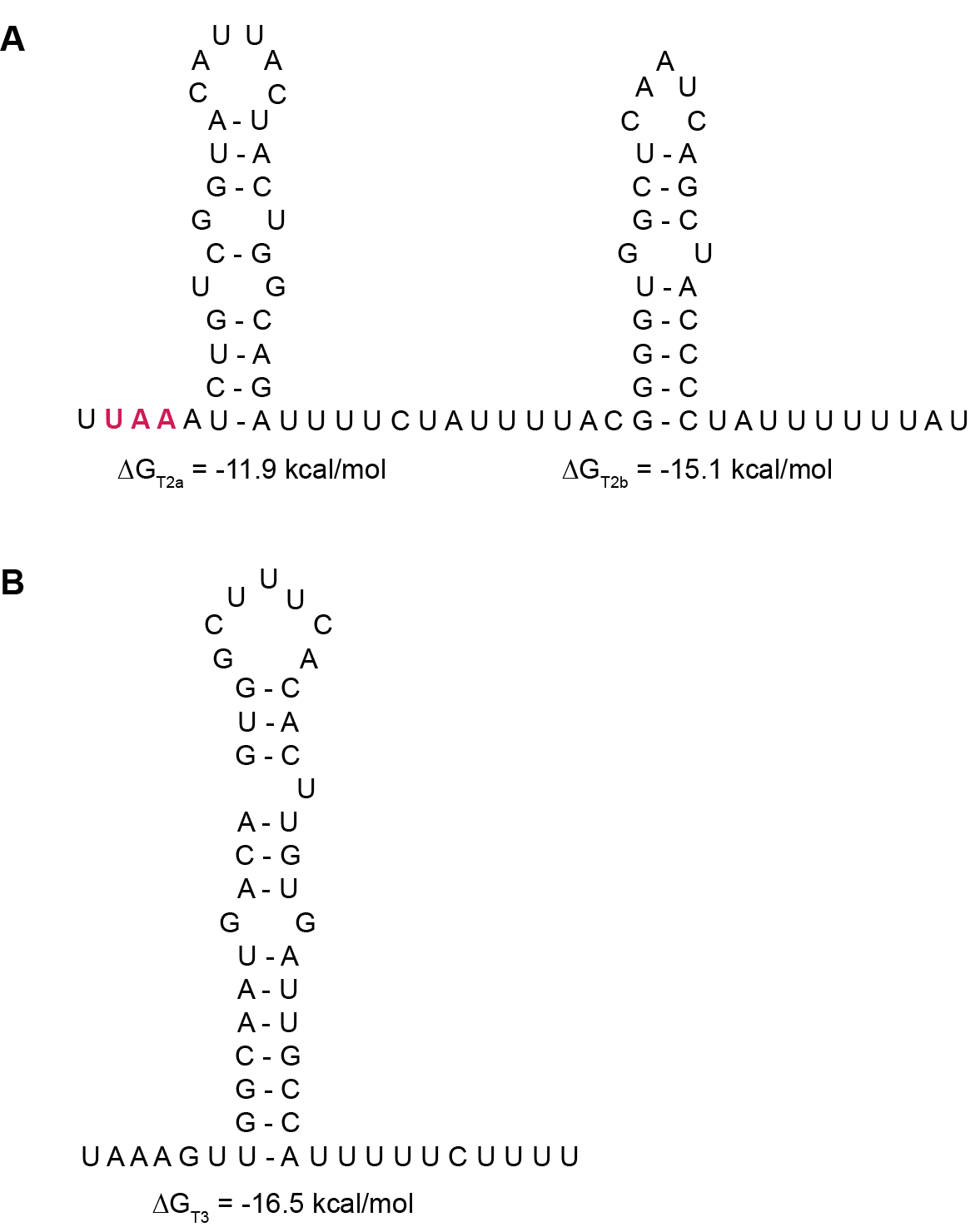

Supplement: S1 Fig — The nucleotide positions of the base of the terminator stem were determined by the ARNold web server [28,29]. The minimum free energy of folding ΔG (of the stem-loop) was calculated using the RNAfold web server [31]. A) Terminator T2a and T2b. Red, bolded UAA indicate the stop codon of orf18. B) Terminator T3. (TIF) [file pgen.1011417.s001.tif]

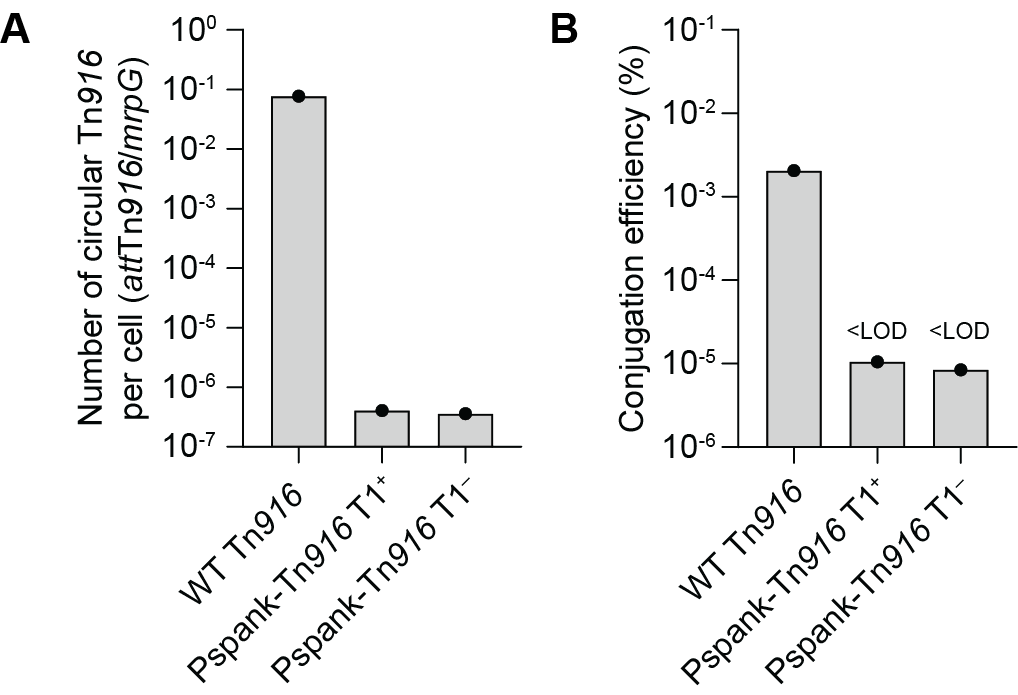

Supplement: S2 Fig — A) Number of circular Tn916 per cell (attTn916/mrpG) and B) conjugation efficiencies of wild-type Tn916 (CMJ253), Pspank-Tn916 T1+ (ESW179), and Pspank-Tn916 T1– (ESW247). All strains were grown without tetracycline. Pspank-Tn916 strains were grown continuously with IPTG. Data presented are from one experiment. Mating assays of Pspank-Tn916 T1+ and Pspank-Tn916 T1– resulted in conjugation efficiencies that are below the limit of detection. (TIF) [file pgen.1011417.s002.tif]

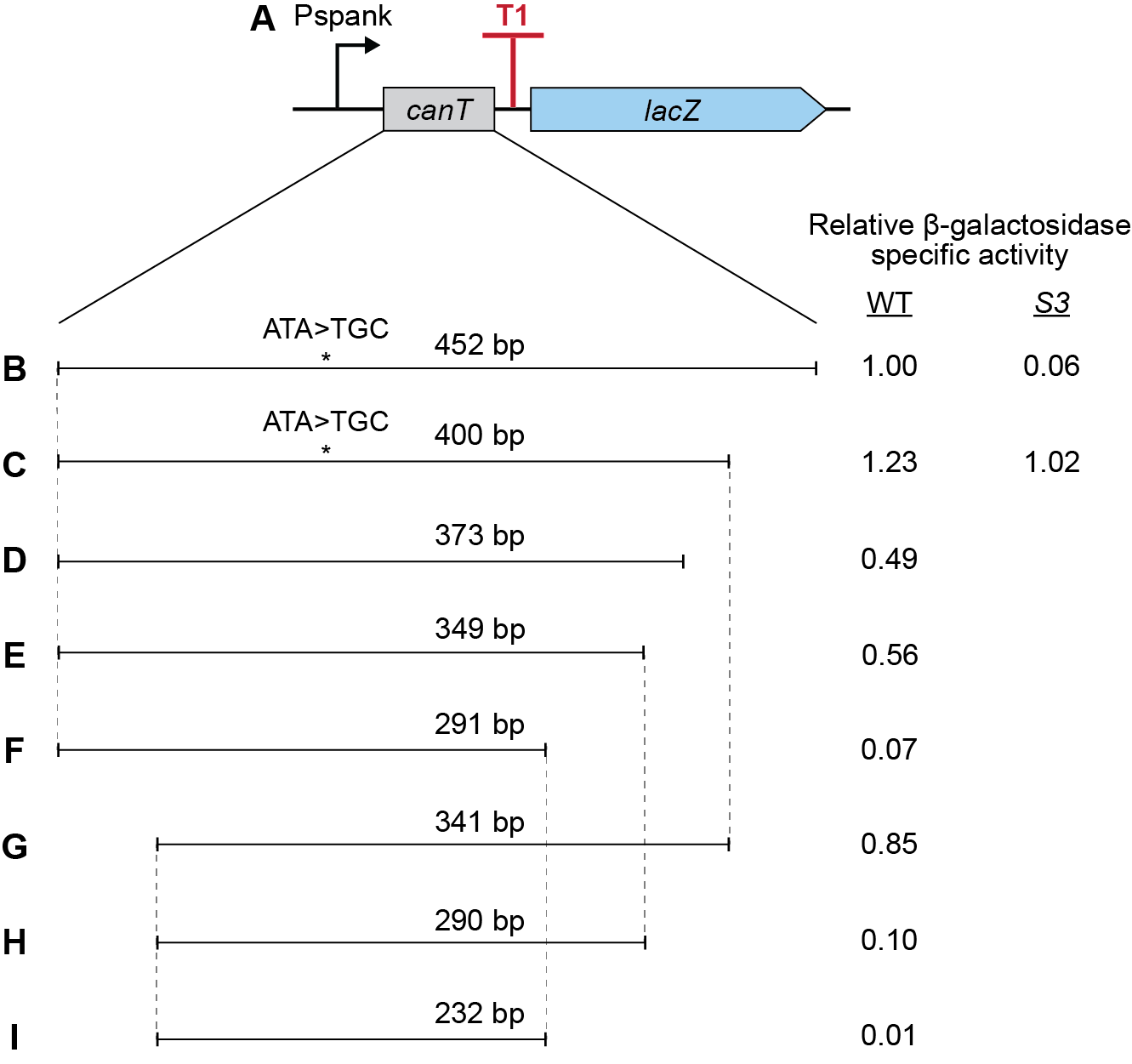

Supplement: S3 Fig — A) Schematic of lacZ reporter construct with canT alleles and terminator T1 between Pspank and lacZ. B-I) DNA regions tested for antitermination activity. The size of each fragment is shown. The effect caused by canT(S3) was determined for two of the cloned fragments (B,C). β-galactosidase specific activities were measured two hours after induction of Pspank with IPTG and relative specific activities were calculated as the mean β-galactosidase specific activity of each strain divided by the mean β-galactosidase specific activity of the strain with the 452 bp fragment of the wild type canT allele. B) [canT (WT, 452 bp), ESW398] and [canT (S3, 452bp), ESW407]. Data for each are from three independent experiments. C) [canT (WT, 400 bp), ESW437] and [canT (S3, 400bp), ESW459]. Data for each are from three independent experiments. D) [canT (WT, 373 bp), ESW450]. Data presented are from three independent experiments. E) [canT (WT, 349 bp), ESW436]. Data presented are from one experiment. F) [canT (WT, 291 bp), ESW422]. Data presented are from one experiment. G) [canT (WT, 341 bp), ESW545]. Data presented are from three independent experiments. H) [canT (WT, 290 bp), ESW438]. Data presented are from one experiment. I) [canT (WT, 232 bp), ESW423]. Data presented are from one experiment. (TIF) [file pgen.1011417.s003.tif]

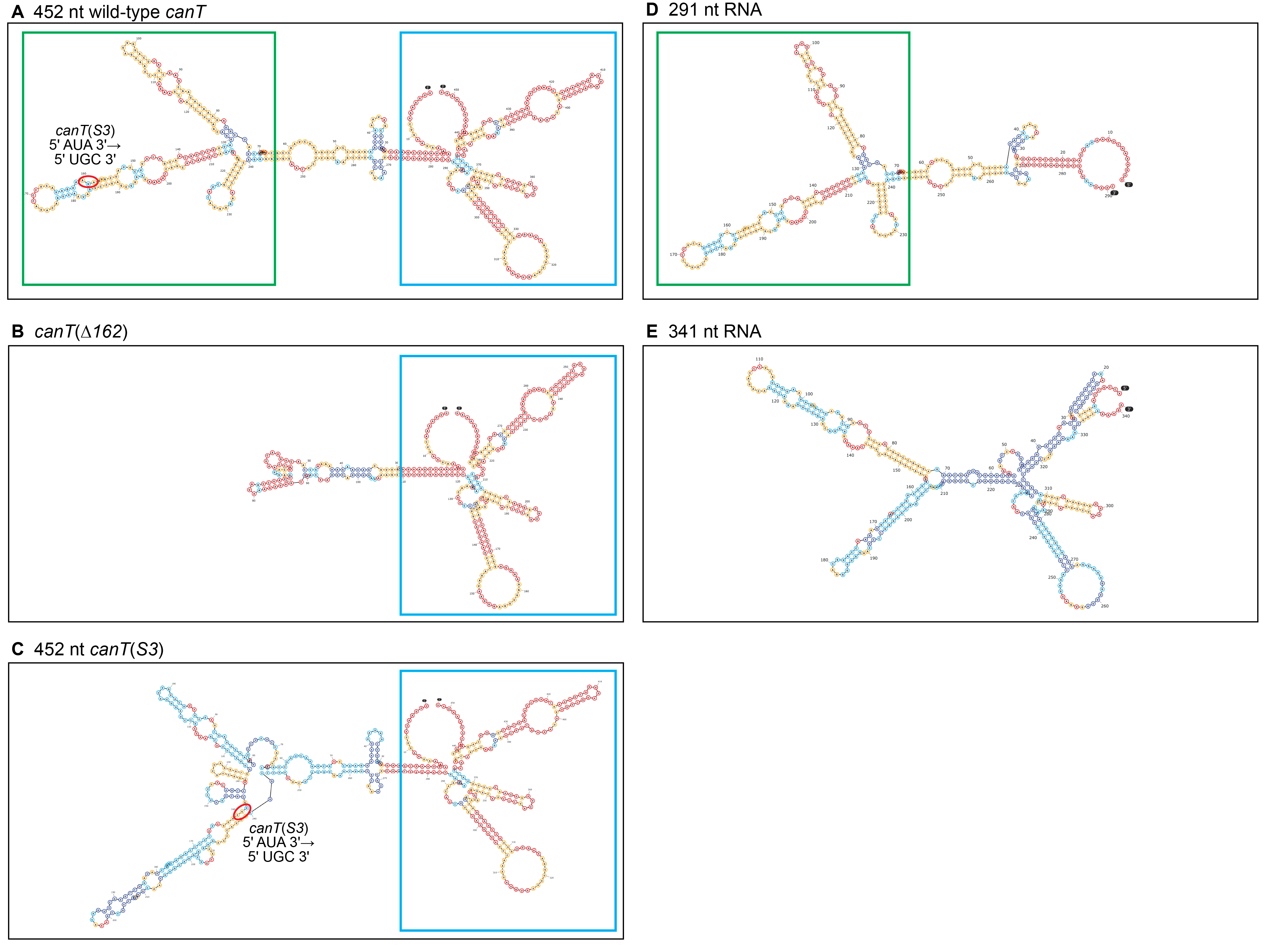

Supplement: S4 Fig — RNA secondary structure predictions were generated using the ViennaRNA package [30] in the SnapGene software. Secondary structures shown are calculated to have the lowest predicted free energy. 5’ and 3’ ends of the RNA are indicated. The predicted structures are colored based on estimated confidence on bases being paired or unpaired (red: 90% and greater, yellow: 70–89%, light blue: 50–69%, dark blue: less than 50%). Structures within green and blue boxes are referred to as the left and right sides of the predicted structures. A) Predicted secondary structure of the 452-nucleotide wild-type canT RNA. The location of S3 mutation is indicated (although the structure is that predicted for the wild-type). B) Predicted secondary structure of the mutant canT(Δ162) RNA. The 5’ and 3’ boundaries are the same as those for the 452-nucleotide wild-type canT RNA, but with 162 nucleotides deleted. See Fig 3B for details on the deletion. This allele is inactive in antitermination. C) Predicted secondary structure of the 452-nucleotide mutant canT(S3) RNA. The canT(S3) mutation changes 3 nucleotides (5’-AUA-3’ to 5’-UGC-3’) as shown. This allele is inactive in antitermination. D) Predicted secondary structure of a 291-nucleotide RNA fragment from the canT region. This allele is inactive in antitermination. E) Predicted secondary structure of a 341 nucleotide RNA fragment from the canT region. This fragment has antitermination activity. (TIF) [file pgen.1011417.s004.tif]

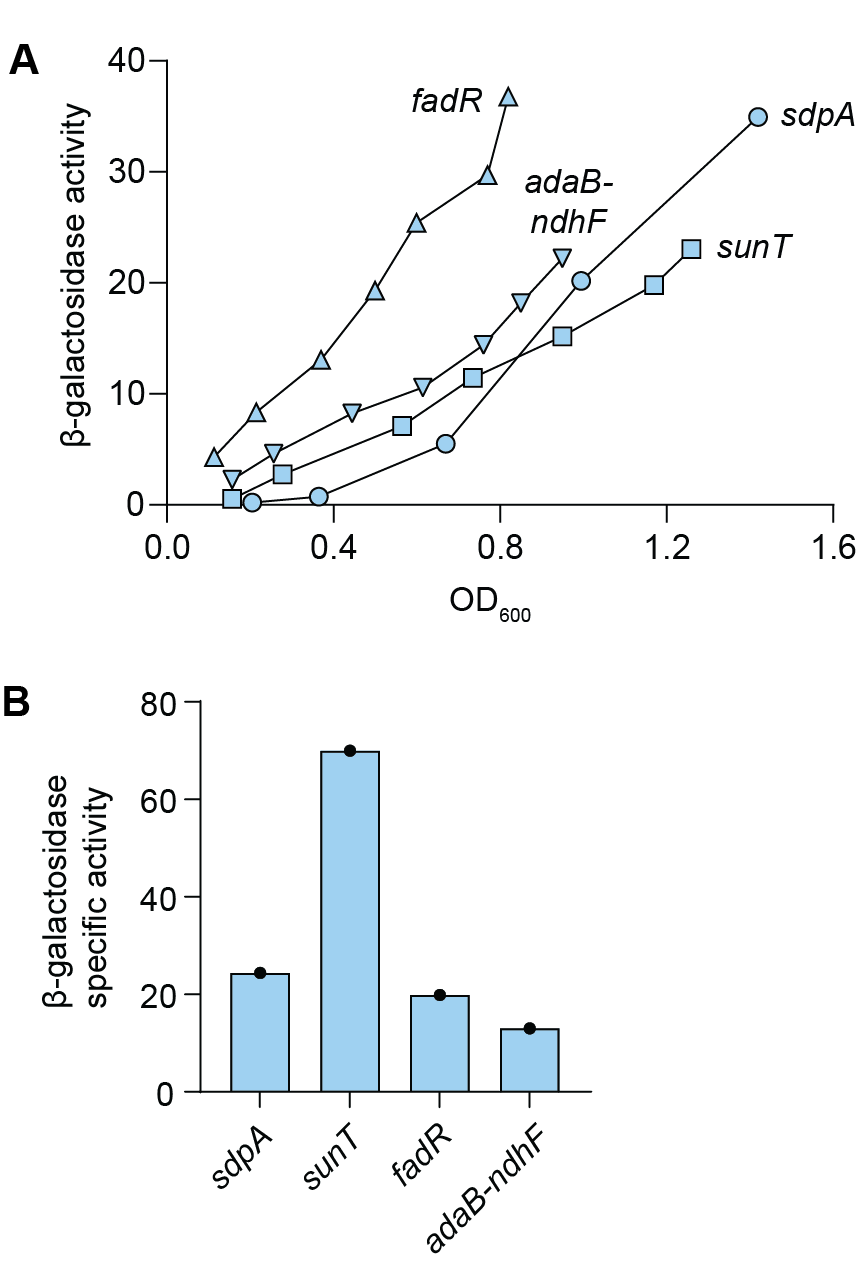

Supplement: S5 Fig — The original Tn916-lacZ insertions were used to monitor expression from host promoters under the conditions indicated. In both panel A and B, data presented are from one experiment. A) β-galactosidase activities are plotted as a function of cell density (OD600) during growth in defined liquid glucose medium at 37°C. The slope of each line is the differential rate of synthesis from the indicated promoter. Circles, sdpA::Tn916-lacZ (ESW517); squares, sunT::Tn916-lacZ (ESW562); triangles, fadR::Tn916-lacZ (ESW557); inverted triangles, adaB-ndhF::Tn916-lacZ (ESW561). B) β-galactosidase specific activities of Tn916-lacZ insertion strains grown as spots on LB agar at 37°C. Measurements were taken from spots resuspended in buffer after 8 hours of growth. Strains used were the same as in panel A. (TIF) [file pgen.1011417.s005.tif]

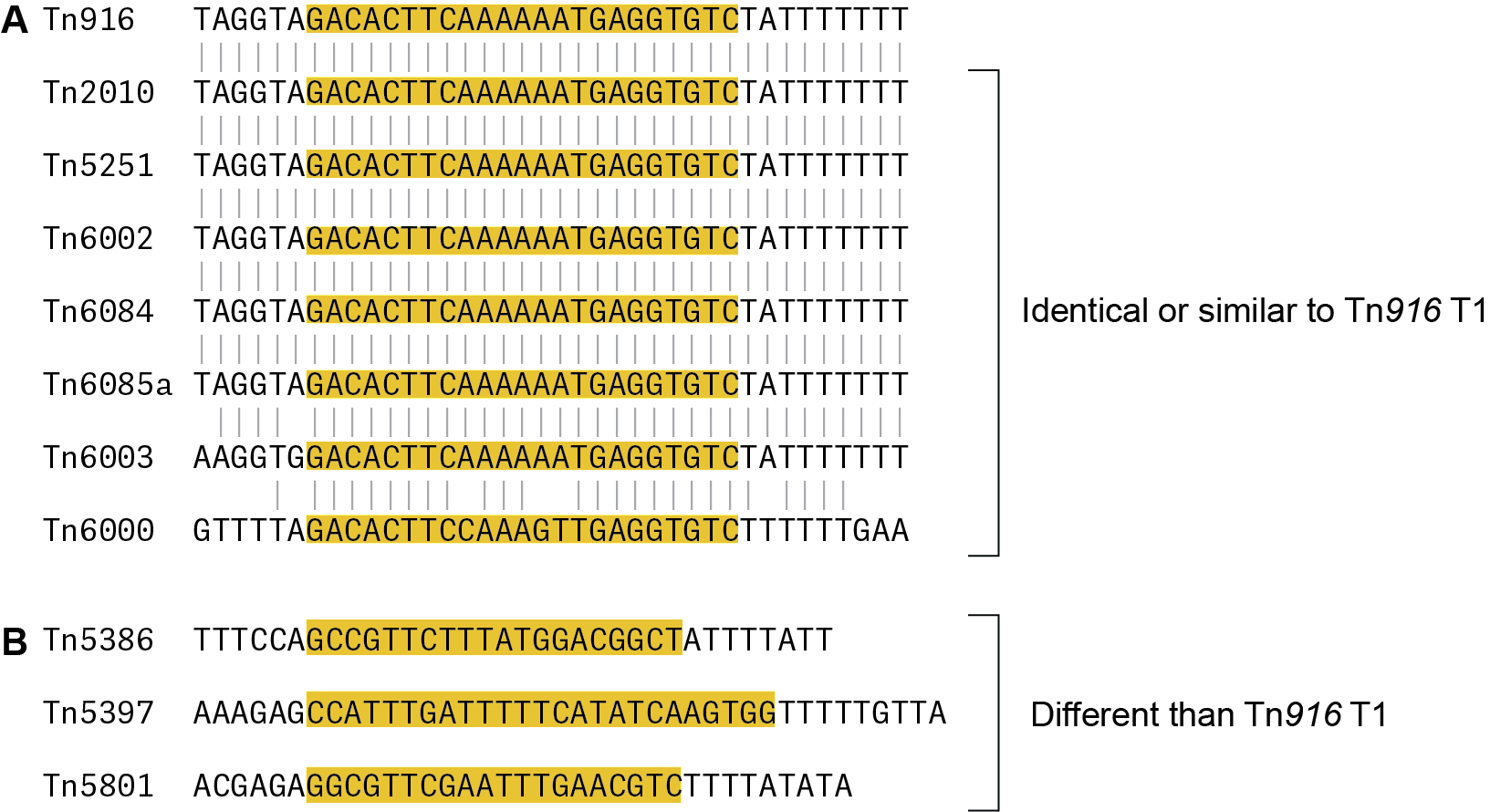

Supplement: S6 Fig — Yellow-highlighted sequences are the predicted stem-loops of the putative terminators near the left end of the indicated elements. None of these have been tested experimentally for terminator activity. A) Sequences that are identical to that of the Tn916 terminator T1 or have a few nucleotide differences in the loop or regions upstream and downstream from the predicted stems. B) Tn5386, Tn5397, Tn5801 have predicted terminators with different sequences than that of Tn916 terminator T1. (TIF) [file pgen.1011417.s006.tif]
